# Evolution of gut microbiota composition from birth to 24 weeks in the INFANTMET Cohort

**DOI:** 10.1186/s40168-016-0213-y

**Published:** 2017-01-17

**Authors:** Cian J. Hill, Denise B. Lynch, Kiera Murphy, Marynka Ulaszewska, Ian B. Jeffery, Carol Anne O’Shea, Claire Watkins, Eugene Dempsey, Fulvio Mattivi, Kieran Touhy, R. Paul Ross, C. Anthony Ryan, Paul W. O’ Toole, Catherine Stanton

**Affiliations:** 1 School of Microbiology, University College Cork, Cork, Ireland; 2 APC Microbiome Institute, University College Cork, Cork, Ireland; 3 Teagasc Moorepark Food Research Centre, Fermoy, Co. Cork, Ireland; 4 Department of Neonatology, Cork University Maternity Hospital, Cork, Ireland; 5 Food Quality and Nutrition Department, Research and Innovation Centre, Fondazione Edmund Mach, San Michele All′adige, Italy

## Abstract

**Background:**

The gut is the most extensively studied niche of the human microbiome. The aim of this study was to characterise the initial gut microbiota development of a cohort of breastfed infants (*n* = 192﻿) from 1 to 24 weeks of age.

**Methods:**

V4-V5 region 16S rRNA amplicon Illumina sequencing and, in parallel, bacteriological culture. The metabolomic profile of infant urine at 4 weeks of age was also examined by LC-MS.

**Results:**

Full-term (FT), spontaneous vaginally delivered (SVD) infants’ microbiota remained stable at both phylum and genus levels during the 24-week period examined. FT Caesarean section (CS) infants displayed an increased faecal abundance of Firmicutes (*p* < 0.01) and lower abundance of Actinobacteria (*p* < 0.001) after the first week of life compared to FT-SVD infants. FT-CS infants gradually progressed to harbouring a microbiota closely resembling FT-SVD (which remained stable) by week 8 of life, which was maintained at week 24. The gut microbiota of preterm (PT) infants displayed a significantly greater abundance of Proteobacteria compared to FT infants (*p* < 0.001) at week 1.

Metabolomic analysis of urine at week 4 indicated PT-CS infants have a functionally different metabolite profile than FT (both CS and SVD) infants. Co-inertia analysis showed co-variation between the urine metabolome and the faecal microbiota of the infants. Tryptophan and tyrosine metabolic pathways, as well as fatty acid and bile acid metabolism, were found to be affected by delivery mode and gestational age.

**Conclusions:**

These findings confirm that mode of delivery and gestational age both have significant effects on early neonatal microbiota composition. There is also a significant difference between the metabolite profile of FT and PT infants. Prolonged breastfeeding was shown to have a significant effect on the microbiota composition of FT-CS infants at 24 weeks of age, but interestingly not on that of FT-SVD infants. Twins had more similar microbiota to one another than between two random infants, reflecting the influence of similarities in both host genetics and the environment on the microbiota.﻿

**Electronic supplementary material:**

The online version of this article (doi:10.1186/s40168-016-0213-y) contains supplementary material, which is available to authorized users.

## Background

The gut microbiota is increasingly regarded as an ‘invisible organ’ of the human body and considered an important factor for host health. This dynamic microbial population develops rapidly from birth until 2 to 3 years of age, when adult-like composition and stability is established [1, 2]. If the establishment of the stable adult microbiota is programmed in infancy, it may lead to a lifelong signature with significant effects on health.

Bacterial colonisation begins at birth, although recent papers have suggested microbiota acquisition occurs in utero [3], challenging the traditional dogma of uterine sterility. The developing gut microbiota of neonates differs widely between individuals [2] and both internal host properties and external factors influence the establishment of the microbiota [4]. At birth, the infant microbial population resembles the maternal vagina or skin microbiota depending on mode of delivery, i.e. by spontaneous vaginal delivery (SVD) or Caesarean section (CS), respectively [5]. Birth mode has a significant effect on the nascent neonatal gut microbiota after these initial founder populations have been replaced [6–9]. At 1 week of age, the microbiota of the SVD infant gut is characterised by high levels of *Bifidobacterium* and *Bacteroides*, while *Clostridium* is more abundant in CS neonates [10]. Numerous other factors have been shown to exert an influence on this development, including antibiotic exposure [11] and breastfeeding [12, 13]. Development of the microbiota occurs as bacteria are replaced in a dynamic, non-random pattern [14, 15]. The use of infant milk formula (IMF) impacts on metabolism [16] and development of the neonatal immune system [17]. This introduction of IMF or solid food perturbs bacterial colonisation [18, 19] and may reduce the benefits of exclusive breastfeeding [17].

Preterm (PT) neonates experience a number of unique challenges to the establishment of their microbiota. CS delivery, maternal and neonatal exposure to antibiotics and the sterile environment of the neonatal intensive care unit (NICU) may all alter the natural pattern of acquisition of microbiota. A few published studies with high subject numbers examining the PT gut microbiota mainly focus on the initial hospitalised period [15, 20]. A knowledge gap surrounding PT gut microbiota development was recently highlighted [21], and to our knowledge, the current study is the largest, well-phenotyped analysis of the longitudinal microbiota development of PT infants after leaving the hospital environment. It has previously been suggested that post-conceptional age, rather than post-birth age, is the main determinant of the bacterial community profile in preterm infants [15]; the aforementioned factors were found to influence the pace, but not the sequence, of microbial acquisition.

Metabolites have been shown to influence regulatory T cells in the gut [22], with changes posited to contribute to autoimmune diseases including inflammatory bowel disease, asthma, allergies, arthritis and multiple sclerosis [23, 24]. These conditions have also been linked to CS and PT birth.

In this prospective study, we compared the gut microbiota of initially breastfed infants from a single geographical area (Cork, Ireland) who were born under different birth modes (SVD or CS) and different gestational ages (FT or PT), in the same maternity hospital. We investigated the effect of both of these factors on the establishment of the nascent gut microbiota of breast fed infants. We also examined the link between the microbiota and the metabolome in early life through comparison of urine metabolomic data with 16S gut microbiota data.

## Methods

### Participants and sample collection

The infants included in this study are part of the INFANTMET study cohort. Mothers were approached for consent between February 2012 and May 2014 at the Cork University Maternity Hospital, with ethical approval provided by the Cork University Hospital Research Ethics Committee (ethical approval reference: ECM (w) 07/02/2012). The study design was to recruit groups of infants according to birth mode and gestation: FT-SVD, FT-CS, PT-SVD and PT-CS infants (PT; less than 35 weeks gestation). Information about the infants was collected at delivery using medical records. Further data were collected using detailed questionnaires given to the mothers when the infants were 1 year old (Additional file 1: Table S1). Faecal samples were collected from the infants at 1, 4, 8 and 24 weeks of age (Table 1). PT infants were sampled at 1 week of age and the same time points (i.e. weeks 4, 8 and 24) after the due delivery date. Samples were collected and placed at 4 °C by the mother, before collection in a temperature-controlled transport collection case by the research nurse for transport to the lab for DNA extraction. An additional sample was acquired at the due date of delivery for PT infants.

**Table 1:** Breakdown of total number of faecal samples collected in the study

	FT-CS	PT-CS	FT-SVD	PT-SVD	Total
Week 1	70	35	83	4	192
Week 4	56	30	63	3	152
Week 8	62	27	74	4	167
Week 24	62	30	74	4	170
Due date	N/A	30	N/A	4	34
Total	250	152	294	19	715

Urine samples were also collected at 4 weeks of age for metabolomic analysis using Sterisets Uricol Urine Collection Pack (Medguard, Ireland). A pad was placed in the diaper and used to collect an unsoiled urine sample from the infant. The pad was then placed in a biohazard bag and frozen immediately by the mothers. This frozen sample was collected in conjunction with the week 4 faecal sample and placed in a −80 °C freezer upon arrival at the lab prior to processing.

The PT infants in the study had a mean gestational age of 31 weeks and 6 days (SD ± 2 weeks 5 days) and mean birth weight of 1715 g (SD ± 564 g). Twenty six of the PT infants were born between 32 and 35 weeks, while the remaining infants were less than 32 weeks gestation (range 24–32 weeks). There were 10 multiple births (9 twin and 1 triplet set) and 20 singleton births; two thirds were male and one third was female. All but four PT infants were born by CS (emergency 73% and elective 12%). The average length of stay in the neonatal unit was 39 days (SD ± 39.14, range 4–190 days). All infants under 32 weeks gestation received one course of antibiotics, with a third receiving at least one additional course. In comparison, only one third of infants born between 32 and 35 weeks gestation received a course of antibiotics and only 4% received a second course. See Additional file 1: Table S2 for further details on PT infants.

### Sample extraction and processing

Faecal samples were processed within 24 h of collection after storage at 4 °C, without freezing. Microbial DNA was extracted from 0.2-g stool samples using the repeat bead beating (RBB) method described by Yu and Morrison [25], with some modifications. A 0.2-g stool sample was incubated with 1 ml RBB lysis buffer (500 mM NaCl, 50 mM tris-HCL, pH 8.0, 50 mM EDTA and 4% sodium dodecyl sulphate (SDS)) in a 2-ml screw cap tube with 0.5 g sterile zirconia beads (A single 3.0 mm bead, 0.1 g of 0.5 mm beads and 0.3 g of 0.1 mm beads). It was homogenised for 90 s (Mini-Beadbeater™, BioSpec Products, Bartlesville, OK, USA), with the tubes cooled on ice for 60 s before another 90 s of homogenisation. Samples were incubated at 70 °C for 15 min to further lyse the cells. Samples were centrifuged (16,000*g*), the supernatant was removed, and the RBB steps were repeated with 0.3 ml of RBB lysis buffer. The supernatants were pooled and incubated with 350 ml of 7.5 M ammonium acetate (SIGMA). The DNA was precipitated by isopropanol, centrifuged at 16,000*g* into a nuclear pellet which was washed with 70% (*v*/*v*) ethanol. The pellet was allowed to dry, then re-suspended in TE buffer, and treated with RNAse and Proteinase K. It was cleaned with QIAGEN buffers AW1 and AW2 using a QIAGEN column and eluted in 200 μl of AE buffer (QIAamp DNA Stool Mini Kit, QIAGEN, UK). DNA was visualised on a 0.8% agarose gel and quantified using the Nanodrop 1000 (Thermo Scientific, Ireland). DNA was then stored at −80 °C.

Primers used for PCR amplification were the V4–V5 region primers 520F (AYTGGGYDTAAAGNG) and 926R (CCGTCAATTYYTTTRAGTTT) (Additional file 1: Table S3). Initial primers for Illumina sequencing contain the sequencing primer binding sites, forward or reverse 16S rRNA gene specific primer and a 10nt in-line multiplexing identifier (MID). Dual separate MIDs were attached to both ends of the PCR product (Additional file 1: Table S3).

The V4–V5 amplicons for Illumina sequencing were generated using a two-step amplification procedure. The first step reaction mix contained 50 μl BIO-X-ACT™ Short Mix (BIOLINE), 10 μl of 2 nM forward and reverse primers, 50 ng genomic DNA and ddH_2_0 to give a final volume of 100 μl. Cycling conditions were the following: an initial 95 °C, 5-min denaturation step; 30 cycles of 95 °C for 15 s, 42 °C for 15 s and 72 °C for 30 s; and a final 10-min extension at 72 °C. The products were purified using SPRIselect beads (Beckman Coulter, Indianapolis, IN) as per manufacturer’s instructions, using a 0.9:1 volume ratio of beads to product. The purified PCR products were eluted in 40 μl of ddH_2_O. DNA quantity was assessed via Quant-iT™ PicoGreen® dsDNA Assay Kit (Invitrogen™). The samples were pooled in equimolar amounts (20 ng DNA per sample) and sequenced at the University of Exeter (UK) using Illumina MiSeq 2 × 300 bp paired-end sequencing, on multiple sequencing runs. Nextflex Rapid library preparation was carried out by the sequencing laboratory to attach bridge adaptors necessary for clustering.

### LC-MS metabolomic analysis of urine

Urine samples were extracted as previously described [26]. A 100-μl urine sample was placed on a 96-well plate with PVDF filter 0.45 μm, together with 200 μl of internal standard in methanol (see Additional file 2: Supplementary materials for details). Samples were then filtered using a positive pressure-96 manifold (Waters, USA). The eluate was diluted with 200 μl of MiliQ water containing cinnamic acid standard. Untargeted LC-MS assays were performed with a hybrid linear ion trap Fourier Transform (LTQ FT) Orbitrap mass spectrometer (Thermo Fisher, Bremen, Germany), in positive and negative ionisation modes. The XCMS Online portal (https://xcmsonline.scripps.edu/) was used for data processing (alignment, peak picking, zero peak re-integrations, features grouping and assessment of quality control samples); please see Additional file 2: Supplementary materials for details. Data obtained from this processing consisted of a list of m/z features and its relative intensities, which vary between sample groups. Such matrix file, with information about sample codes, m/z feature and its intensity, was used for statistical analysis. In positive ionisation mode, 2380 statistically significant features were found. In negative ionisation mode, there were 3832 statistically significant features. To annotate compounds, a selection strategy was used based on the most abundant and the most statistically significant features. The procedure for annotation of compounds was adapted from standard metabolomic initiatives (see Additional file 2: Supplementary materials for details). Levels of identification were as follows: level I corresponds to compounds identified by matching masses and retention times with authentic standards in the laboratory, or by matching with high-resolution LC-MS and LCMSn spectra of standards reported in the literature; and level II corresponds to compounds identified by matching with high- and low-resolution LC-MS and LC-MSn spectra from databases and literature. Compounds identified only by spectral similarities to a similar compound class and literature knowledge are reported as level III. Unknown compounds are reported as level IV.

### Bioinformatic analysis

The Illumina MiSeq 2 × 300 bp paired-end sequencing reads were joined using the Fast Length Adjustment of SHort reads to improve genome assemblies (FLASH) programme [27]. MIDs were extracted and sequences were assigned to their corresponding individual samples by QIIME’s split_libraries_fastq.py, permitting two ambiguous bases per MID (Ns), and using QIIME’s default quality settings. The USEARCH sequence analysis tool [28] was used for further quality filtering. Sequences were filtered by length, retaining sequences with lengths of 350–370 bp. This range was used to select the most abundant sequences for the base of each operational taxonomic unit (OTU) with reads of all lengths then aligned to the OTU sequences. Single unique reads were removed, and the remaining reads were clustered into OTUs. Chimaeras were removed with UCHIME, using the GOLD reference database. The original input sequences were mapped onto the OTUs with 97% similarity. All reads were taxonomically classified by the classify.seqs command within the mothur suite of tools (v1.31.2), using the RDP reference database (training set 14) [29]. OTUs were classified from these when >50% of the reads agreed on a classification at each phylogenetic level. The returned read numbers varied greatly from 129 to 815,400 reads (average = 69,410 reads per sample). To adjust for the influence of the number of sequences in a sample on diversity and other statistical tests, any sample with less than 10,000 sequence reads was eliminated from the study. This resulted in the loss of eight samples from the data set. Fifteen samples had been sequenced in duplicate, so the samples with the lower read numbers of duplicated pairs were removed, as we believed that these may not be the best representations of those samples due to the lower read counts. The OTU table containing the remaining 715 samples was rarefied to 10,000 reads, to remove any bias from variation in sample read numbers. The remaining samples were from variable modes of delivery and time points (data not shown).

### Culture-dependent analysis

One gramme of fresh faecal sample per infant was serially diluted in maximum recovery diluent (Fluka, Sigma Aldrich, Ireland). Enumeration of bifidobacteria was performed by spread-plating serial dilutions onto de Man, Rogosa, Sharpe agar (Difco, Becton-Dickinson Ltd., Ireland) supplemented with 0.05% L-cysteine hydrochloride (Sigma Aldrich), 100 μg/ml mupirocin (Oxoid, Fannin, Ireland ) and 50 units nystatin suspension (Sigma Aldrich). Agar plates were incubated anaerobically at 37 °C for 72 h (Anaerocult A gas packs, Merck, Ocon Chemicals, Ireland). Enumeration of lactobacilli was determined by plating samples onto *Lactobacillus* selective agar (Difco) with 50 units nystatin and incubated anaerobically at 37 °C for 5 days. Bacterial counts were recorded as colony forming units (CFU) per gram of faeces and were log10 transformed prior to statistical analyses.

### Statistical analysis

Statistical analysis was performed using the R statistical framework, using a number of software packages or libraries including, made4, vegan, DESeq2, car, nlme and lme4. Relative abundance bar charts were generated with Microsoft Excel. Where possible, statistical analyses of changes over time take the subject numbers into account, such as the alpha diversity linear modelling, and DESeq2 tests for differential abundance.

To assess alpha diversity, we calculated the Shannon Diversity Index with the diversity function from the R vegan package. After fitting Shannon Diversity to multiple distributions and performing Shapiro-Wilk normality tests, we found that it best approximated a normal distribution, as determined by Quantile-Quantile plots (qqplots; not shown). Therefore, differences of alpha diversity between infants of different modes of delivery at a given time were detected using mixed effect linear models (R package nlme), which allow for the adjustment of sequencing run (random effect), while testing for differences due to mode of delivery (mixed effect). In order to compare alpha diversity over time, mixed effect linear models were applied (R package lme4, and Analysis of Deviance using the ANOVA command from the Car package to test for significance), which allow for controlling for the subjects and the age of the infants, along with sequencing run.

Multiple beta diversity metrics were also calculated, including weighted and unweighted UniFrac and Spearman distance ((1 – Spearman Correlation)/2). Principal coordinates analysis was performed on each beta diversity metric to highlight the separation of infants based on mode of delivery and sampling time point. Differences between groups were tested for using permutational multivariate analysis of variance (PerMANOVA) on beta diversity matrices, adjusting for sequencing run. False discovery rate was adjusted for with Benjamini-Hochberg [30].

To identify taxa (phyla and genera) that may be driving the significant differences detected between time points and mode of delivery, differential abundance analysis was determined using DESeq2 on raw phylum- and genus-level count data. We determined that DESeq2 was an appropriate tool for differential abundance analysis as the negative binomial model best fit all genera, determined by the “goodfit” command from the “vcd” R package. A heatplot was generated to highlight the major genera driving clustering of samples from different modes of delivery at different time points and to identify bacterial co-abundance. We used only genera that were found in at least 10% of the samples, and utilised Spearman correlation and Ward clustering on log10 of the rarefied genus count matrix.

We determined significant differences of culture-dependent count data between time points or mode of delivery using the Wilcoxon rank sum test, and adjusted for false discovery rate with Benjamini-Hochberg. Correlations between culture-dependent (plate count) and culture-independent (16S sequencing) data were determined using Pearson’s product-moment correlation. Pearson’s product-moment correlation was also used to determine if abundance of any genera correlated with that of any other genera, and the false discovery rate was adjusted with Benjamini-Hochberg. To determine if twins were more closely related to each other than random infants, we performed *t* tests with Monte-Carlo simulations on beta diversity between samples.

The urine metabolomics dataset was unit scaled before significant features were identified using the ANOVA statistical test with term and delivery mode as explanatory variables. This analysis gave consistent results when compared to pareto scaled data and ANCOVA as the statistical test with 84% of the identified metabolites being returned (data not shown). The logged fold change and the mean value for each variable were calculated and the results were filtered using the false discovery rate (FDR) calculated from the raw *p* values. To aid the identification of metabolites, an additional clustering analysis was performed by WGCNA cluster analysis using the Spearman correlation and a soft threshold of nine [31].

## Results

### Drivers of infant gut microbiota

#### Gut microbiota is influenced by mode of delivery and gestational age

**Fig. 1:**
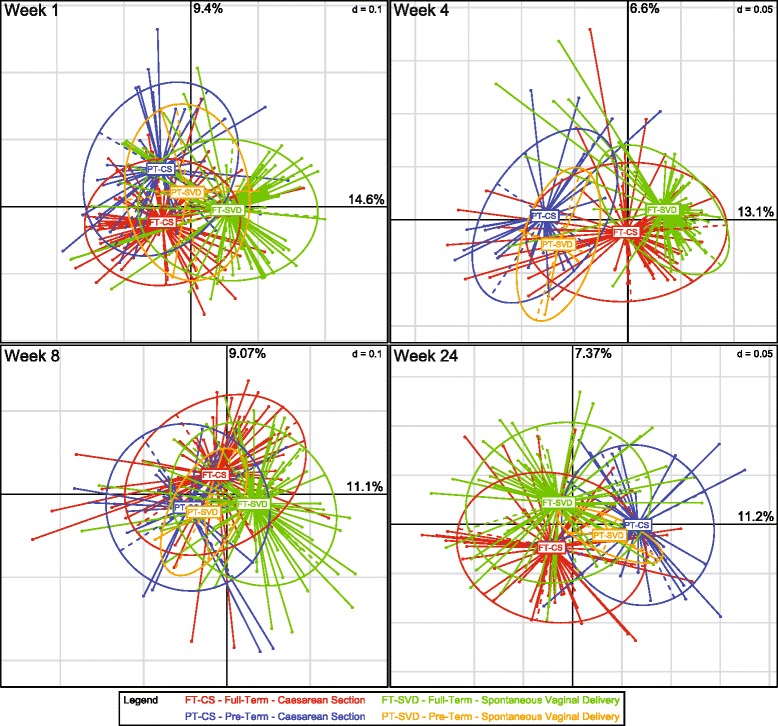
Birth mode and gestation age both significantly affect the composition of the infant gut microbiota to 24 weeks of age. Principal coordinates analysis (PCoAs) on Spearman distance matrices of samples at each of four time points (weeks 1, 4, 8 and 24) revealed significant differences between the groups. Significance was calculated using permutational multivariate analysis of variance (PerMANOVA, Additional file 1: Table S3). *p < 0.05; **p < 0.01; ***p < 0.001

The structure of the infant gut microbiota is clearly affected by mode of delivery (Fig. 1, Additional file 1: Table S4). The results demonstrate that there was a significant difference in microbiota composition at genus level across the four different groups from week 1 to week 24, when analysed by Spearman distance matrix and visualised by principal coordinates analysis (PcoA). At 1 week of age, the microbiota composition of the FT-CS group was significantly different from that of both PT-CS and FT-SVD groups (*p* values <0.001). PT-CS and FT-SVD were also distinct from one another (*p* < 0.001). The low number of PT-SVD infants (*n* = 4) did not permit significant testing at this time point, but it is worth noting that this microbiota cluster is situated between the PT-CS and FT-SVD groups. At 4 weeks of age, FT-CS microbiota was significantly different to all other groups (*p* < 0.001). The PT infant microbiota mainly separated across the x-axis. At 8 weeks of age, the FT-CS group is distinct from both PT-CS and FT-SVD (*p* < 0.001), separated on both axes. The FT-SVD and PT-CS are also distinct (*p* < 0.001); all three groups have significantly different microbiota composition at 8 weeks of age. By 24 weeks, there were no significant differences between PT-CS and FT-CS microbiota, while FT-CS and FT-SVD microbiota were still significantly different (*p* < 0.01). At this time point, mode of delivery remains influential while differences due to gestational age have been eliminated. At all time points, there was wide diversity of individual population structures within each group, showing the heterogeneous composition of the developing infant gut microbiota.

#### Distinctive metabolomic profiles are associated with microbiota profiles

**Fig. 2:**
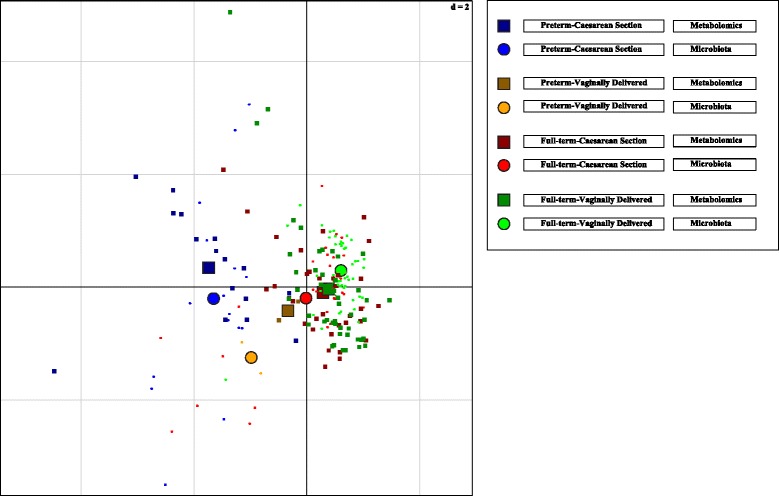
Co-inertia analysis of urine-derived metabolomic and 16S rRNA gut microbiota data from stool. Microbiota data was scalar normalised and logged. Microbiota is represented by *circles* and the metabolomic samples are represented by *squares*. Four groups are visualised; preterm-Caesarean section (*blue*), preterm-spontaneous vaginal delivery (*orange*), full-term Caesarean section (*red*) and full-term spontaneous vaginal delivery (*green*). Small objects represent the individual samples and large objects represent the barycentre of the group. Analysis shows that the co-variance between the microbiota and metabolomics dataset splits the preterm infants from the full-terms. Metabolites associated with this split are highlighted in Additional file 3: Figure S1 and Additional file 1: Table S4

Co-inertia analysis of the week 4 microbiota data at the OTU level and the metabolomic dataset showed that there was a significant (*p* < 0.05) amount of co-variation in the two datasets (Fig. 2). There was little separation observed between FT birth modes (FT-CS and FT-SVD); however, the PT-CS samples separated distinctly from the FT samples. The co-inertia analysis showed that there were greater differences between the group microbiota profiles than between the group metabolomic profiles. These differences are evident where the FT metabolomic baricentres are overlaid while there is a separation between the microbiota baricentres. The PT-SVD metabolomic and microbiota baricentres were relatively distant from one another, but this separation may be due to the low number of samples in each of these groups. The compounds associated with the PT-FT split are from multiple different sources and were represented by a diverse selection of metabolites (Additional file 3: Figure S1, Additional file 1: Table S5). Annotated metabolites were grouped based on their origin and chemical character: (i) amino acids and metabolites; (ii) carboxylic acids and phenolic acids and their metabolites; (iii) vitamins and their metabolites; (iv) drugs and their metabolites; (v) carnitines; (vi) indole metabolites; and (vii) fatty acids and their metabolites (see Additional file 1: Tables S5 and S6). Urea and its associated metabolite derivatives were situated in the centre of the metabolite cluster, suggesting it is abundant in both groups, providing confidence in our classifications.

We found a number of paracetamol metabolites to be significantly higher in PT infants, as well as several different vitamins and their metabolites such as riboflavin, CECH—a tocopherol metabolite or pyridoxic acid (Additional file 1: Table S5). These metabolites may be due to altered medical treatment of PT infants. Among endogenous metabolites found to be statistically significant, two families could be easily identified: tryptophan and tyrosine. Metabolites belonging to tryptophan pathway were kynurenine, indoxyl sulphate, indole acetic acid, while those belonging to tyrosine were acetylphenylalanine, acetyl tyrosine and hydroxyphenylalanine sulphate.

The accurate mass and fragmentation pattern of a number of features that were elevated in the urine of the PT group were consistent with bile acids; all of them conjugated to glycine. We found glycocholic acid, one sulphate conjugate of chenodeoxyglycocholic acid (or its isomer glycoursodeoxycholic acid) and three atypical bile acids (Additional file 1: Table S5).

Among small carboxylic acids we found succinic acid and its derivatives are statistically lower in PT, while several metabolites of glutamic acid were found at higher levels.

Finally, a number of fatty acids were found to be statistically higher in PT-CS infants. Most of them were found as dicarboxylic species, with different hydroxylation patterns and with different saturation levels; moreover, some were found as glucuronide conjugates.

#### Differentially abundant taxa drive microbiota clustering over time

**Fig. 3:**
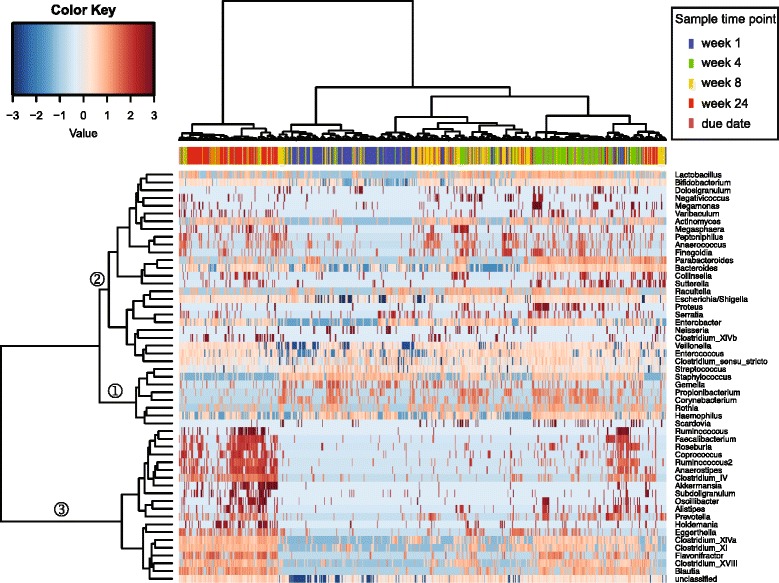
Infants separate temporally and into three distinct clusters based on differentially abundant taxa. The three clusters may indicate the beginning of an enterotype-based microbiota profile as early as 24 weeks of age. Only those genera (*side*) that are present in at least 10% of samples (*top*) are shown. Samples are highlighted by the time point at which they were obtained

The abundance of genera can also be represented by a heat plot of hierarchically clustered samples (Fig. 3), which helps classify differentially abundant taxa. Many of the samples cluster by time point, where week 1 and week 24 in particular show relatively tight clusters. Weeks 4 and 8 show some variation, which is possibly due to the inclusion of FT and PT infants, whereas PT infants are slightly older, due to the time point of sampling. Very few genera are present at high abundance at week 1, as bacterial diversity is lowest at this time point. The genera that are abundant at week 1 decrease in relative abundance by week 24 (branch 1), as other genera begin to emerge at detectable levels as the infants age (branch 2). This trend is visible through week 8, when another cluster of genera begins to emerge (branch 3). By week 24, the genera that were most abundant at week 1 have markedly reduced in proportion (branch 1). Genera on branch 2 have low relative abundances, whereas genera on branch 3 are found to be quite highly abundant. Within this third branch we find genera that are core to enterotypes, such as *Prevotella*, *Blautia* and *Ruminococcus*. Once *Bifidobacterium* emerged (at week 1 for some samples, and by week 4 for others), their abundances appear to remain relatively stable, at least to 24 weeks of age.

#### Breastfeeding influences the gut microbiota of CS infants

**Fig. 4:**
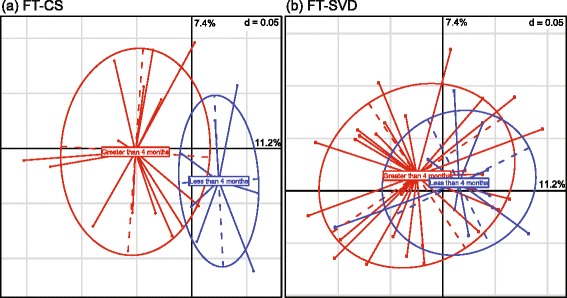
Breastfeeding duration influences the gut microbiota of C-section infants but not naturally delivered infants at 24 weeks of age. **a** Caesarean section, full-term infants. **b** Naturally delivered full-term infants. In *blue* are infants that were breastfed for less than 4 months (i.e. between 1 and 2 months, or between 2 and 4 months). In *red* are infants that were breastfed for longer than 4 months. The vast majority of infants in the cohort were breastfed for 1 month

We collected categorical metadata on how long each infant was breastfed. Three categories were recorded: between 1 and 2 months, between 2 and 4 months, and greater than 4 months. We used PerMANOVA to compare the microbiota of infants in these categories, and also examined the birth mode effect separately (Additional file 1: Table S7). No differences were detected between the microbiota composition of infants who were breastfed for 1 to 2 months and those breastfed for 2 to 4 months. However, comparing infants breastfed for less than 4 months and those for longer than 4 months revealed a significant difference for FT-CS but not FT-SVD (Fig. 4). Five genera were significantly more abundant in infants that were breastfed for longer and four genera were more abundant in infants that were breastfed for a shorter duration (Additional file 1: Table S8). *Bifidobacterium* was not found to significantly differ in abundance based on duration of breastfeeding (also tested with Wilcoxon Rank Sum test; data not shown).

#### Twins have more similar gut microbiota than unrelated infants

There were ten sets of twins and one set of triplets within the cohort. Twenty one of these 23 infants were in the PT-CS category; we therefore focussed only on these infants. Using *t* tests with Monte-Carlo permutations, we determined that at week 1, twins’ microbiota were more similar within twin pairs than between non-twin pairs (Spearman distance test: *p* < 0.001). This is also true at weeks 4, 8 and 24 (*p* < 0.001 at each time point) (Additional file 4: Figure S2).

#### Gut microbiota of preterm infants is influenced by post-birth age

For the week 1 time point, all infants were approximately 1 week post-birth (range 6 to 8 days). However, at other time points, PT infants were chronologically older than FT infants, as the samples for these time points were collected at weeks post due date rather than post-birth. This was to ensure all infants were the same post-conceptional age when sampled, as this was previously postulated to have the greatest effect on the establishment of the microbiota [15]. Infants were assigned to groups depending on how many weeks premature they were at birth (4, 5, 6, 7, 8, 9 or 12 weeks). We found that at 1 week of age, when all PT infants are the same post-birth age, no significant difference or trends were apparent. At predicted due dates, three comparisons showed significant differences, with a further seven showing a trend for differences (Additional file 1: Table S9).

### Description of the infant gut microbiota

#### Differential abundance at phylum and genus level in infant groups

We utilised DESeq2 in order to identify bacteria responsible for the microbiota separation of the different groups at phylum and genus levels. The main differences are outlined below, with a full list of all differentially abundant genera available in Additional file 1: Tables S10–S17).

#### Phylum level

**Fig. 5:**
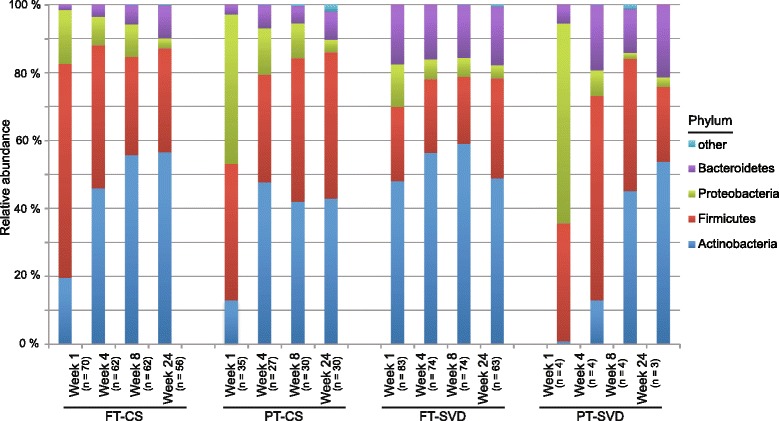
Naturally delivered infant microbiota remains stable at phylum level from 1 to 24 weeks of age, while C-section delivered infants progress to a similar microbiota profile over time. There is no shift in the FT-SVD infant composition from 1 to 24 weeks of age. FT-CS progresses by increasing the relative abundance of Actinobacteria (*p* < 0.001) and Bacteroidetes (*p* < 0.001) and decreasing the relative abundance of Firmicutes (*p* < 0.05) over the same period. PT-CS infants initially have a higher abundance of Proteobacteria compared to the FT groups (*p* < 0.001). Between week 1 and week 4 the Proteobacteria and Firmicutes abundance decreased (*p* < 0.001 and *p* < 0.01, respectively). No significant differences were recorded after week 4. The PT-SVD group had low subject numbers (*n* = 4), hindering significant associations, resulting in no significant changes being observed. Showing phyla found at >1% average in total population. Phyla found at <1% were grouped as ‘other’

Using relative abundance of phyla from the rarefied dataset, we determined the major bacterial phyla in the different infant groups (Fig. 5). A clear distinction is apparent between the different infant groups at 1 week of age. The FT-SVD infants have a relatively consistent microbiota composition from 1 to 24 weeks of age. The dominant phylum throughout this period is the Actinobacteria (mainly comprised of the genus *Bifidobacterium).* FT-CS infants initially had a higher relative proportion of Firmicutes at 1 week of age compared to FT-SVD (*p* < 0.05) and less Actinobacteria (*p* < 0.001). At 4 weeks of age, Actinobacteria (*p* < 0.01) and Bacteroidetes (*p* < 0.001) were more abundant in FT-SVD infants, again with Firmicutes less abundant (*p* < 0.01). Within the FT-CS group, Actinobacteria significantly increased in relative abundance from 1 to 4 weeks of age (*p* < 0.001). Bacteroidetes increased significantly in proportion from week 4 to week 8 (*p* < 0.05) and again from week 8 to week 24 (*p* < 0.001). Thus, the FT-CS microbiota progressed over time to one which is similar to the FT-SVD infants with no differences at phylum level at either 8 weeks or 24 weeks of age.

The most pronounced difference between the FT-CS infant gut and the PT-CS gut is evident at 1 week of age when there is a significantly higher proportion of Proteobacteria in PT infants (*p* < 0.001). The PT-CS group also harbours an initially high relative proportion of Firmicutes at week 1 before becoming dominated by both Actinobacteria and Firmicutes from weeks 4 to 24.

#### Genus level

**Fig. 6:**
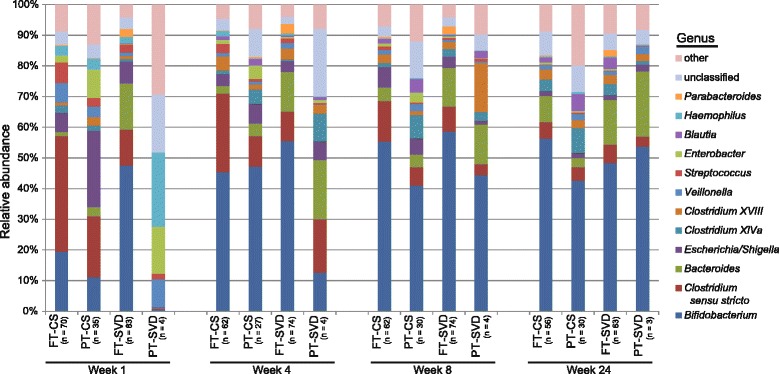
Comparison of the microbiota composition of infants born by different birth modes and gestation duration at the same age across four time points from 1 week to 24 weeks of age. The most pronounced differences are evident at week 1 of age, with the microbiota composition becoming increasingly uniform over time to 24 weeks. Showing genera found at >1% average in total population. Genera found at <1% were grouped as ‘other’

The infant gut is dynamic and a number of genera demonstrate significant abundance differences between groups (Fig. 6 and Additional file 1: Table S10–S13) and within groups at different ages (Additional file 1: Table S14–S17). We identified genera which were differentially abundant in at least two of the groups and thus found 21 genera that had dissimilar abundances at week 1, 41 genera at week 4, 39 genera at week 8 and 25 genera at week 24 (Additional file 1: Tables S10–S13). Some of the significant changes are described below.

As expected, *Bifidobacterium* were found to be a major component of the infant gut. *Bacteroides* and *Clostridia* were also important contributors to the gut microbiota composition. Despite the apparently large difference in the average proportion of *Bifidobacterium* at week 1 between FT-CS and FT-SVD (19 vs 48%), this difference is not statistically significant, due to the high inter-individual variation between infants at this early age. There was no statistically significant difference in the relative proportion of *Bifidobacterium* between these two birth modes at any time point. *Bacteroides* was found to be significantly more abundant in FT-SVD infants compared to FT-CS at both 1 and 4 weeks of age (*p* < 0.001), but not at later time points. *Parabacteroides* were also significantly more abundant at 4 weeks of age in FT-SVD than FT-CS (*p* < 0.001). We also observed that PT-CS had a higher relative proportion of *Bacteroides* at 4 weeks of age compared to FT-CS. *Clostridium sensu stricto* is significantly more abundant in FT-CS when compared to FT-SVD at week 1 (*p* < 0.001). At this time point, it is also more abundant in PT-CS than PT-SVD (*p* < 0.01) but, as for *Bacteroides*, no differences were observed at the later time points.

We noted very little development of the FT-SVD microbiota composition at genus level throughout the 24 week period studied (Fig. 6, Additional file 1: Table S16). From week 1 to 4 to 8, no genus was found to be significantly altered in relative proportion. From week 8 to week 24, only *Blautia* (*p* < 0.05), and *Subdoligranulum* (*p* < 0.01; representing a very low proportion of the overall microbiota composition) were found to significantly change in abundance. Contrastingly, there were 12 and 16 genera that showed significantly altered relative proportions between week 1 and week 4 for FT-CS (Additional file 1: Table S14) and PT-CS (Additional file 1: Table S15) respectively, indicating a high level of microbiota change in the early life of these infants. Sampling of subsequent time points does not demonstrate this level of compositional change indicating a stabilisation of the infant gut microbiota. The statistical analysis of PT-SVD gut microbiota development was hindered by low subject numbers, but notwithstanding this limitation, we observed a number of significant changes between week 8 and week 24, with relatively few changes at earlier time points (Additional file 1: Table S17).

Co-abundance analysis is a useful way to reveal higher-level constraints and associations in microbiota composition [32]. We therefore examined the relationship between abundances of genera. Given that many genera appeared or increased in abundance over the time period assessed within this study we expected numerous positive correlations. We therefore tested whether any genera were negatively correlated. Of 12,090 correlations tested, after adjusting for multiple testing, 16 were significant negative correlations (Additional file 1: Table S18). Fourteen of these significant negative correlations involved *Bifidobacterium*.

#### Diversity of the gut microbiota increases with age

**Fig. 7:**
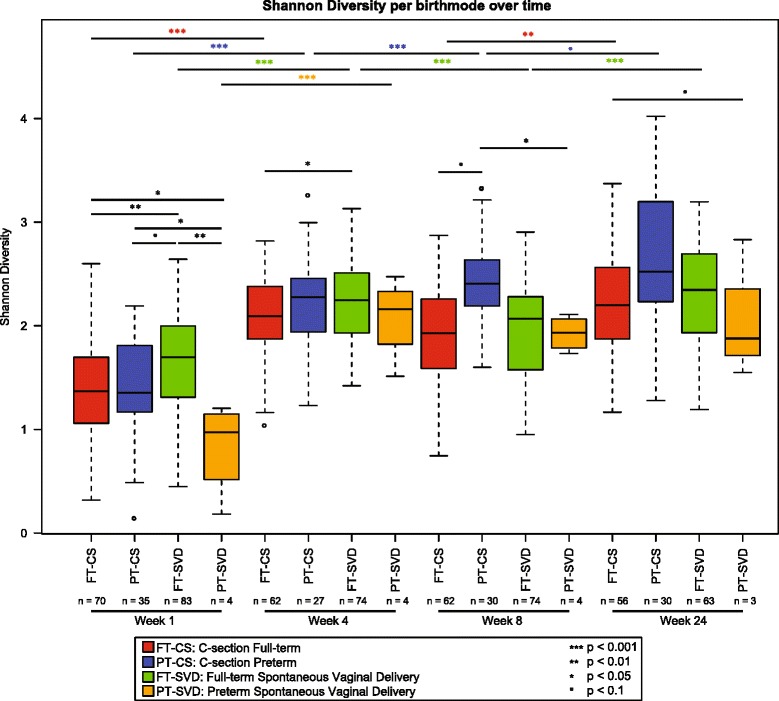
Shannon diversity of different groups of infant gut microbiota increases with age, demonstrated by separating subjects by both age and by birth mode. Significant differences between birth modes at a given time point were tested with a linear mixed effects model which adjusts for potential batch effect (sequencing run), and the age of the infants at the given time point. Comparing different time points for a given birth mode was performed with linear mixed effects models that adjust for the batch and the subjects

Alpha diversity is a measure of the overall diversity of the community present in a sample. The alpha diversity of the infant gut microbiota was shown to be influenced by age and birth mode (Fig. 7). As measured by the Shannon index, α-diversity increases as the infant ages. However, the microbiota diversity in each group did not increase equally over the first 24 weeks of life (Fig. 7, Additional file 5: Figure S3). We observed that the groups have a different diversity relative to each other at week 24 when compared to week 1. At 1 week of age, the FT-SVD microbiota displays the highest diversity of all groups, while PT-SVD is lowest. Diversity of all four groups increases from week 1 to week 4. FT-SVD increases in diversity between 1 and 4 weeks of age (*p* < 0.001); then diversity reduces slightly between week 4 and week 8 (*p* < 0.001); before increasing again between week 8 and 24 (*p* < 0.001). PT-CS diversity increased from weeks 1 to 4 (*p* < 0.001); 4 to 8 (*p* < 0.001); and showed a trend toward an increase from week 8 to 24 (*p* < 0.1). FT-CS increased from weeks 1 to 4 (*p* < 0.001) and weeks 8 to 24 (*p* < 0.01) but not between weeks 4 and 8. The overall increase in bacterial diversity is comparable in both FT groups. PT-SVD infants microbial diversity increased between week 1 and 4 (*p* < 0.001) and then appears to stabilise, with no further significant increase at 24 weeks. By week 24, there are no significant differences in alpha diversity between any groups, but a trend can be observed for higher diversity in FT-CS over PT-SVD, suggesting that infants of different birth modes and term have reached an equivalent diversity state at this age.

#### Correlation of bacterial culture and culture-independent data

**Fig. 8:**
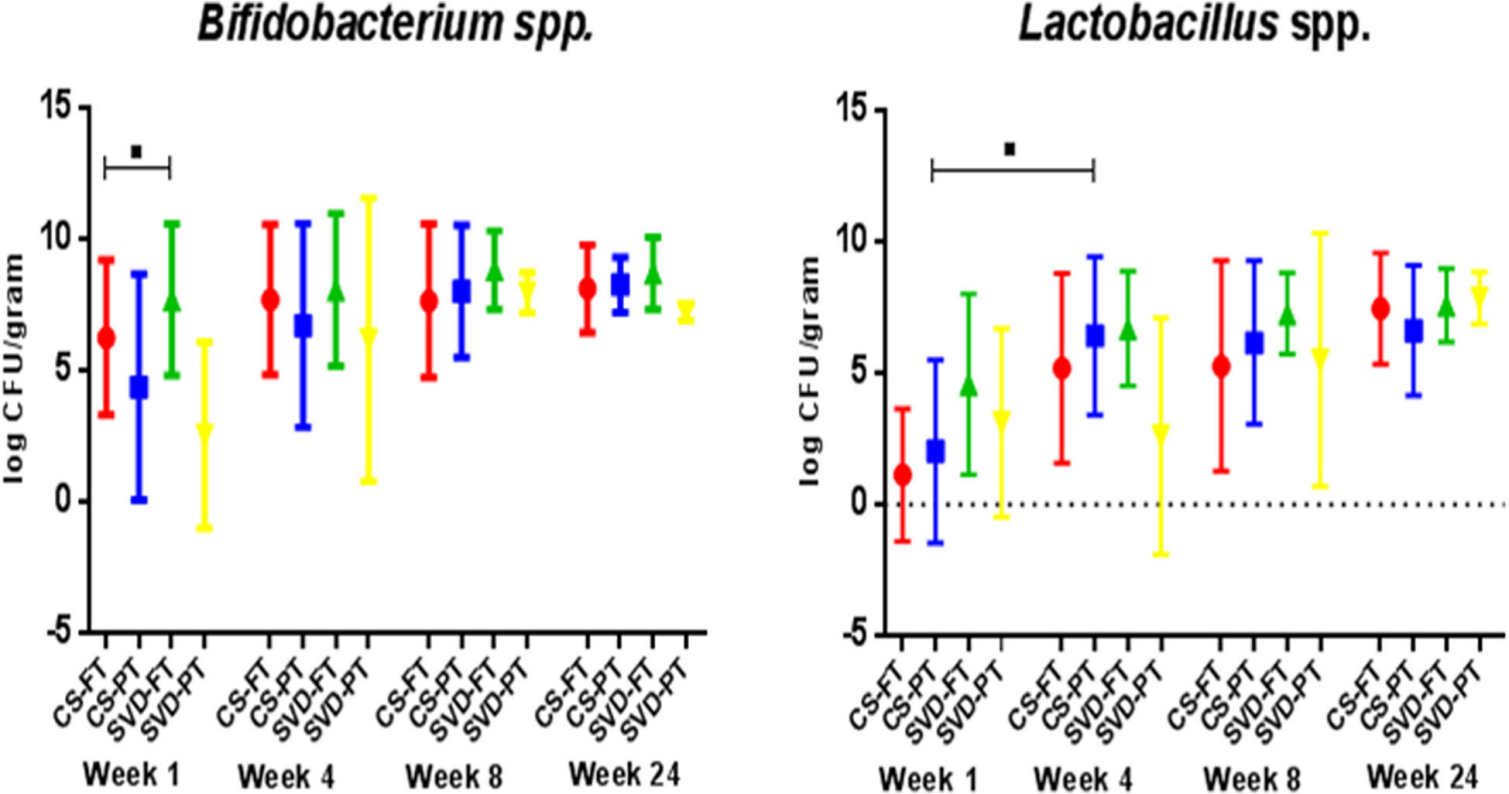
Count data showing the absolute levels of *Bifidobacterium* and *Lactobacillus* at all time points from 1 to 24 weeks of age for all four infant groups. Culture techniques were used to generate count data to verify the accuracy of the culture-independent sequencing data. Over 7000 strains of Bifidobacterium and Lactobacillus stocks were isolated and stocked in a biobank

To isolate representative strains of major taxa, we also performed bacterial culture on the infant faecal samples. *Bifidobacterium* and *Lactobacillus* bacterial populations in infant faeces were determined by plate counting (Fig. 8). We observed a significant correlation between the *Bifidobacterium* levels recorded by 16S analysis, and the colony forming unit (CFU) counts (*ρ* = 0.24; *p* value = 6.64e−07). Likewise, *Lactobacillus* counts correlated with 16S-based data (*ρ* = 0.21; *p* value = 1.96e−05).

To determine whether significant differences in *Bifidobacterium* or *Lactobacillus* counts were present between birth modes at a given time point, or between time points for a given birth mode, we applied the Wilcoxon rank sum test with Benjamini-Hochberg adjustment. Due to the high level of variation recorded, we only observed a trend for higher *Bifidobacterium* counts in FT-SVD over FT-CS at week 1 (Fig. 8). *Bifidobacterium* counts at week 1 were highest in FT-SVD (mean 7.45 log CFU/g) and lowest in PT-SVD infants (mean 2.57 log CFU/g). Bifidobacteria numbers increased across all groups from week 1 to week 4, while at week 8, counts were observed to stabilise. At week 24, numbers remained stable across all groups. Similarly, *Lactobacillus* was also most abundant in FT-SVD (mean 3.98 log CFU/g) and lowest in PT-CS infants (mean 1.12 log CFU/g) at week 1. Counts increased in all groups (except PT-SVD) from week 1 to week 4; from week 4 to week 8 counts were stable in the PT-CS, FT-CS and FT-SVD groups, while an increase was observed in PT-SVD infants.

## Discussion

In this prospective study, we compared the gut microbiota of a cohort of initially breastfed infants from a single geographical area (Cork, Ireland) who were born under different conditions; SVD or CS delivery modes, either FT or PT, in parallel with clinical data to 2 years. Here, we present the data obtained in the first 24 weeks of life. Our results consolidate and corroborate recent findings surrounding the importance of birth mode on the initial development of the microbiota [6, 33–35], with robust data ensured by the large number of subjects. The infants were sampled at four time points across the first 24 weeks of life to enable longitudinal analysis of the development of the nascent gut microbiota and were breastfed for a minimum of 4 weeks to ensure there was a standard initial feeding regime. We found a significant effect of PT-CS birth on infant microbiota compared to FT infants, most likely due to a number of challenges unique to PT infants, including feeding type, antibiotic exposure (maternal and infant), increased duration of hospital stay, gut immaturity and immune immaturity. Low numbers of PT-SVD infants were recruited as very few infants fitting to our definition of PT (<35 weeks gestation or <1500 g) were born via SVD delivery mode in the hospital where the study was based. Although it would be interesting to observe if PT infants not subjected to the environmental and antibiotic exposures detailed above still had an atypical microbiota profile in early life, such a prospective study would be very difficult to perform because these medical interventions are clinically necessary for PT infant health. The current study accurately represents the results of typical conditions encountered by PT infants in developed countries. It should be noted that by week 24, there are no differences in alpha diversity between any of the groups. This may indicate that the microbiota has recovered from the stresses associated with birth mode to a large extent by 6 months of age. Alternatively, the view could be expressed that this is a crucial developmental window for the establishment of lifelong host health.

The urine-derived metabolome was shown by co-inertia analysis to mirror separations observed in the faecal microbiota data at 4 weeks of age. This strongly suggests that the microbiota of the infants is modulating the metabolites produced. The specific metabolites driving the separation between FT and PT infants were diverse in nature, with the PT-CS gut in particular having a large amount of altered metabolites. These results are in good agreement with the study of Hyde et al. [36] who studied birth mode in piglets, wherein CS piglets were found to have lower concentration of succinic acid, and higher concentration of glutamic acid than SVD piglets. They also collate with Dessi et al. [37], which examined urine of infants by nuclear magnetic resonance. In this study, the most important discriminating compounds were associated with tyrosine, proline and arginine metabolism, as well as with biosynthesis of tryptophan, tyrosine, phenylalanine and the urea cycle. At the systems level, regulation of tryptophan, tyrosine and phenylalanine pathways plays a role in gut-brain signalling. A microbial influence on the profile of metabolites within these pathways is suspected to contribute to diverse psychiatric and behavioural disorders including autism, schizophrenia and depression [38–40].

Bile acid derivatives are also present in greater amounts in the PT infant urine metabolome, which may be indicative of either jaundice or differential action of the microbiota in the gut of the infant. Strandvik et al. [41] found tetrahydroxylated atypical bile acids were predominant in PT newborns. Bile acids are main by-product of cholesterol metabolism in the liver, and play a major role in maintaining bile flow. Many enzymes are involved in the conversion of cholesterol into bile acids; thus, improper functioning of one of them can result in formation of atypical bile acids, and probable liver dysfunction [42]. It is not known for how long a time after birth this difference persists, thus the significance and physiologic effects require further investigation.

The occurrence of dicarboxylic fatty acids suggests the expression of alternative fatty acid oxidation mechanisms. Considering the presence of phenylacetylglycine among biomarkers, which is associated with disorders of mitochondrial fatty acid beta-oxidation, we can suggest that in PT infants, lipid metabolism was affected. This was also suggested by the aforementioned study by Hyde et al., where CS piglets accumulated more hepatic lipid than their SVD counterparts. A series of liver free fatty acids were also found to be up-regulated in CS piglets.

Phthalates are associated with hospital stays [43], PVC flooring [44] and formula feeding and are more abundant in the PT metabolome. Paracetamol derivatives were also at higher levels in the PT gut, and are found in commonly used medical products, such as Calpol^®^. Carnitine is associated with red meat and has been controversially suggested as being linked to increased risk for cardiovascular disease [45]; levels of carnitine were found to be elevated in the PT gut. However, vitamin D and vitamin E were also more abundant in the PT metabolome, demonstrating that not all metabolites observed as elevated in the PT urine are traditionally viewed as unfavourable metabolites. This alteration of the gut environment as a neonate is clearly a potential mode for progression of the conditions which are associated with PT birth. More work is needed to establish if the microbiota is a causative agent of this altered metabolomic profile, and if modulation of the infant gut microbiota would restore the metabolomic profile of a PT infant to that of a FT infant.

There was a strong temporal separation of the infant gut microbiota, showing close grouping together by age, particularly for the samples at week 1 and week 24 (Fig. 3). The infants were divided into three separate clusters based on differential abundance of certain taxa. A large number of genera were absent at early time points and then present at a relatively high level by 24 weeks of age, forming a distinct cluster. This cluster contained genera that are core in adult enterotypes, such as *Prevotella*, *Blautia* and *Ruminococcus*. This observation of the emergence of enterotype-like groupings of infants provides a strong foundation to suggest that a lifelong microbiota signature may be imprinted upon infants as early as at six months of age. Alpha diversity, as measured by the Shannon Diversity Index, increased (or remained at the same level) with age across all groups except from week 4 to week 8 in FT-SVD infants. We were unable to attribute this anomaly to any particular factor, and it remains an interesting outlier in the dataset.

We observed an initially high proportion of Actinobacteria (almost exclusively *Bifidobacterium*) in FT-SVD, Firmicutes in FT-CS and Proteobacteria in PT infants at 1 week of age. The observed differences in microbiota composition were most pronounced at this early age. The diversity of the PT gut microbiota at this time is low, which may be due to maternal and neonatal antibiotic treatment. At genus level, *Bacteroides* were found to be significantly less abundant at both 1 and 4 weeks of age in FT-CS infants compared to FT-SVD infants (both *p* < 0.001); *Parabacteroides* are also significantly reduced in the FT-CS infants compared to FT-SVD at 4 weeks of age (*p* < 0.001). This indicates that Bacteroidetes may play a major role alongside *Bifidobacterium* in the early infant gut microbiota, especially as certain species of *Bacteroides* can metabolise human milk oligosaccharides [46]*.*

At 4 weeks of age, a trend was observed whereby these breastfed FT-CS infants’ gut microbiota gradually developed to one more closely resembling that of FT-SVD infants. At 8 weeks of age, the FT-CS microbiota continued to become more similar to that of FT-SVD infants, while PT infant community microbiota profile remained more distinct. We noted that twins had a significantly more similar microbiota composition than two unrelated infants, which was anticipated. At this age, twins have recently shared the same womb, birth mode (typically CS), and large portion of their genetic makeup, in addition to breastfeeding and environment. Therefore, the microbiota of these individuals would be expected to be more similar and it would be interesting to observe how long this similarity lasts as the infants’ age. In a recent study by our group, investigating the gut microbiota composition in dichorionic triplets, we observed that host genetics initially appeared to play a significant role in the composition of the individual gut microbiota, but by 12 months environmental factors were the major determinant [47].

Although the microbiota of FT-CS and PT infants is similar at genus level at 24 weeks of age, it remains distinct at OTU level. The differences in the gut microbiota during this critical developmental period of an infant’s life may have a long lasting effect on the immune system or intestinal barrier function [48–51]. It is also known that the microbiota has an influence on the metabolic capabilities of an individual [52, 53]. This would place CS, particularly for PT infants, firmly into a high risk category for a number of developmentally associated disorders and diseases. Early-life perturbations of the developing infant gut microbiota have also recently been shown to impact on the central nervous system via the gut-brain axis and may potentially lead to adverse mental health outcomes later in life [54].

We observed a significant effect of breastfeeding on the microbiota of the FT-CS infants but not on that of the FT-SVD infants. Infants born by CS lack the natural inoculum of bacteria obtained during passage through the birth canal received by SVD infants. Instead, they are exposed to environmental bacteria initially rather than vaginal bacteria. These results show that FT-CS infants may benefit from breastfeeding by obtaining some of the bacteria that they are initially lacking because of birth mode. SVD infants, contrastingly, have a “natural” microbiota profile that is not as obviously further developed by exposure to breast milk, at least in terms of alterations at genus level. *Bifidobacterium* levels have previously been shown to be promoted by breast feeding [12, 55] but though present at a high level in the infants in our cohort, it was not one of the genera which increased in abundance in breast fed infants (Additional file 1: Table S8). It is very likely that the high proportion of breast feeding in the first month of life of the infants in this cohort has lessened or masked to some extent the impact of sustained breastfeeding, particularly in terms of maintaining the initial bacterial profile established in FT-SVD infants.

Had post-conceptional age played an important role in shaping the gut microbiota of infants, we would have expected their microbiota to be more similar at their due dates, where all should theoretically be the same post-conceptional age, and different at week 1, when they were at different post-conceptional ages. However this was not observed. We found that the infants’ microbiota composition were more similar at week 1, when they were all the same age after birth, and were different at their due dates, when they would have different “post-birth” ages at this point. This shows that age since birth is more important than post-conceptional age, but that neither exerts a large influence on shaping the composition of the PT neonatal microbiota.

We also noted that although certain bacteria, such as *Haemophilus*, have a low relative proportion of the overall microbiota (average relative abundance = 1.4%), they have a very high prevalence (75% of all infants) in the INFANTMET cohort. This suggests they are occupying a small, specific niche or carrying out a specific function as part of the functioning ecosystem of the developing gut. We also observed *Bifidobacterium* involved in a number of negative correlations with other genera, which is unsurprising as it becomes a more dominant component of the FT-CS, PT-CS and PT-SVD gut microbiota over time. This shows the competitive advantage that *Bifidobacterium* has in the environment of the infant gut as it displaces other bacteria.

Further novelty was added to this study through the use of culture-dependent analysis in conjunction with culture-independent sequencing. Culture-dependent techniques remain valuable for their ability to isolate bacterial species for further analysis. We focussed on populations of two bacterial groups, *Bifidobacterium* spp. and *Lactobacillus* spp., as these are known natural inhabitants of the healthy infant intestinal microbiota, and there is substantial evidence demonstrating the role of these bacteria as potential probiotics [56–58]. Indeed, a bank of over 7000 putative *Bifidobacterium* and *Lactobacillus* strains derived from infant faecal samples from INFANTMET is currently being screened for probiotic potential. Substantial inter-individual variation was observed for *Bifidobacterium* and *Lactobacillus* counts which supports findings from other studies of infant intestinal microbiota [59, 60].

More research is needed to establish whether one factor, or a combination of antibiotic treatment, gestation length, C-section birth, or formula feeding regime are causally associated with autoimmune and metabolic diseases. However, the evidence from this study and the current literature certainly suggests that factors which disturb the establishment of the neonatal microbiota should be avoided or minimised as much as possible. Future studies may focus upon best methods for restoration of the natural composition of the neonatal gut following perturbation by any of the aforementioned factors. While probiotics are one avenue with some potential, the effects will likely be strain specific. Combination studies, in conjunction with dosage testing, will need to be tightly regulated in any prospective intervention.

## Conclusions

The data indicate that mode of delivery and gestational age at birth are the strong influencers of early gut microbiota structure following birth, with the impact of mode of delivery and gestational age still apparent 24 weeks after birth, although diminished with age. Currently, little is known in literature about the overall metabolic status of term and preterm neonates, and only a limited number of metabolites are routinely measured in their biological fluids by conventional methods. Co-inertia analysis of (urine) metabolomic and (gut) microbiota data indicate that they correlate with one another, showing a functional influence of the altered microbiota on several metabolic pathways of PT-CS infants in particular.

Infants separated temporally by differentially abundant taxa. The PT gut has a number of unique initial challenges at birth resulting in a distinct initial composition at 1 week of age, high in Proteobacteria (*p* < 0.001). There was very little development of the FT-SVD microbiota composition throughout the first 24 week of life, with FT-SVD infants retaining a relative stable microbiota composition over this period, while FT-CS infants develop a similar composition to FT-SVD after 8 weeks of age. Of particular note was the prevalence of Actinobacteria and Bacteroidetes in FT-SVD infants very early in life but that the FT-CS and PT infant gut microbiota “catch up” in the first 24 weeks. *Bifidobacterium* was found to be a major component of the infant gut, but not significantly impacted by duration of breast feeding. Prolonged breastfeeding (>4 months) was found to impact the gut microbiota of FT-CS infants but not FT-SVD infants.

*Bacteroides* were significantly more abundant in FT-SVD infants compared to FT-CS infants over the first month of life indicating an important role, perhaps similar to the importance currently attributed to *Bifidobacterium* in the early development of the infant gut microbiota.

Taken together, these data highlight the importance of breastfeeding particularly for infants born via Caesarean delivery mode and the data provide new opportunities for optimisation of IMF composition, with appropriate new bioactive ingredients such as milk fractions, probiotics and prebiotics to effectively programme the early infant gut microbiota in a manner closer to mother’s milk.

Best practice currently would appear to confirm the adage that ‘breast is best’, and all current evidence appears to validate that statement for the maintenance of a healthy neonatal gut microbiota, and this is particularly true for infants delivered by CS birth mode.

## Additional files

Additional file 1: Table S1. Metadata collected from the Infantmet cohort. Blank space in the metadata table indicates that information on that particular factor was not received for that infant (or mother). **Table S2.** Preterm infant details and clinical metadata, including gestational age and antibiotic exposure(s). **Table S3.** Complete list of all oligonucleotides used for PCR amplification. **Table S4.** The influence of birth mode and getational age on the infant gut microbiota from week 1 to 24 of life. Results of pairwise PerMANOVA tests on Spearman distance matrix between birth modes at given time points, after adjustment for batch effects. *p* value adjustment was performed using Benjamini-Hochberg. **Table S5.** Reporting classes of metabolites annotated in urine. **Table S6.** 2S MS2 and MS3 spectra of annotated biomarkers. Detailed breakdown of the classification of the biomarkers used for metabolomics analysis. **Table S7.** Prolonged breastfeeding influences FT-CS but not FT-SVD infants’ gut microbiota. PerMANOVA *p* value results comparing the microbiota of infants breastfed for different durations, after adjusting for potential batch effect. Where birth modes were combined, *p* values were obtained after adjusting for birth modes. **Table S8.** Genera found to be significantly different at week 24 between infants that were breastfed for less than, or greater than 4 months. In red (positive log2 fold change) indicates genera that are higher in infants that were breastfed for longer, whereas in green are genera that are lower in infants that were breastfed for longer. **Table S9.** Pairwise comparisons of Spearman distances between groups of infants at due dates, grouped by the number of weeks early that they were born. PerMANOVA was used on Spearman distance matrix between all samples of the group pairs being tested, and multiple testing. **Table S10.** Genera found to be differentially abundant, or trending toward significance, between birth modes at week 1. L2FC: Log2 fold change; p.adj: adjusted *p* value; sig.: significance level. Positive L2FC (in blue) indicates that the genus is higher in the first birth mode of the pair listed, whereas a negative *p* value (in orange) indicates that the genus is lower in the first birth mode listed. **Table S11.** Genera found to be differentially abundant, or trending toward significance, between birth modes at week 4. L2FC: Log2 fold change; p.adj: adjusted *p* value; sig.: significance level. Positive L2FC (in blue) indicates that the genus is higher in the first birth mode of the pair listed, whereas a negative *p* value (in orange) indicates that the genus is lower in the first birth mode listed. **Table S12.** Genera found to be differentially abundant, or trending toward significance, between birth modes at week 8. L2FC: Log2 fold change; p.adj: adjusted *p* value; sig.: significance level. Positive L2FC (in blue) indicates that the genus is higher in the first birth mode of the pair listed, whereas a negative *p* value (in orange) indicates that the genus is lower in the first birth mode listed. **Table S13.** Genera found to be differentially abundant, or trending toward significance, between birth modes at week 24. L2FC: Log2 fold change; p.adj: adjusted *p* value; sig.: significance level. Positive L2FC (in blue) indicates that the genus is higher in the first birth mode of the pair listed, whereas a negative *p* value (in orange) indicates that the genus is lower in the first birth mode listed. **Table S14.** Genera found to be differentially abundant, or trending toward significance, between time points for FT-CS. L2FC: Log2 fold change; p.adj: adjusted *p* value; sig.: significance level. Positive L2FC (in blue) indicates that the genus is higher at the earlier of the two compared time points, whereas a negative *p* value (in orange) indicates that the genus is lower in the first of the two time points. **Table S15.** Genera found to be differentially abundant, or trending toward significance, between time points for PT-CS. L2FC: Log2 fold change; p.adj: adjusted *p* value; sig.: significance level. Positive L2FC (in blue) indicates that the genus is higher at the earlier of the two compared time points, whereas a negative *p* value (in orange) indicates that the genus is lower in the first of the two time points. **Table S16.** Genera found to be differentially abundant, or trending toward significance, between time points for FT-SVD. L2FC: Log2 fold change; p.adj: adjusted *p* value; sig.: significance level. Positive L2FC (in blue) indicates that the genus is higher at the earlier of the two compared time points, whereas a negative *p* value (in orange) indicates that the genus is lower in the first of the two time points. **Table S17.** Genera found to be significantly different, or trending toward different, between time points for PT-SVD. L2FC: Log2 fold change; p.adj: adjusted *p* value; sig.: significance level. Positive L2FC (in blue) indicates that the genus is higher at the earlier of the two compared time points, whereas a negative *p* value (in orange) indicates that the genus is lower in the first of the two time points. **Table S18.** Significant negative correlations of abundances of genera pairs. Correlations were tested with Pearson correlation. The false discovery rate was adjusted for with Benjamini-Hochberg adjustment. (XLSX 188 kb)

Additional file 2: Supplementary Materials and Methods. (DOC 39 kb)

Additional file 3: Figure S1. A selection of specific metabolites driving the split in the co-inertia analysis that separates FT and PT gut by their metabolomic profile. Each metabolite is represented by one or more features. The metabolites on the left hand side are higher in PT infants and the metabolites on the right hand side are higher in FT infants. (PDF 21785 kb)

Additional file 4: Figure S2. Twins have more similar microbiota to one another than an unrelated infant at 1, 4, 8, and 24 weeks of age. Using *t* tests with Monte-Carlo permutations, the Spearman distance between twins’ microbiota was determined and compared to unrelated infants’ microbiota (***p < 0.001). (PDF 138 kb)

Additional file 5: Figure S3. Assessing the magnitude of change of Shannon diversity between time points for subjects of a given birth mode. Statistical models were used to determine if infants of one birth mode changed more or less than infants of other birth modes at different stages. Grey asterisks highlight significant differences from linear mixed effects models that adjust for batch effect and the number of days between time points for each subject. Black asterisks highlight significant differences as determined by linear mixed effects models, adjusting for batch effect, the number of days between time points, and the age of infants at the first time point. (TIF 638 kb)

## Abbreviations

CS: Caesarean section
FLASH: Fast Length Adjustment of SHort reads to improve genome assemblies
FT: Full term
IMF: Infant milk formula
LC-MS: Liquid chromatography–mass spectrometry
MID: Multiplexing identifier
NICU: Neonatal intensive care unit
OTU: Operational taxonomic unit
PCoA: Principal coordinates analysis
PT: Preterm
RBB: Repeat bead beating
SDS: Sodium dodecyl sulphate
SVD: Spontaneous vaginal delivery

## References

[1] Maynard CL, Elson CO, Hatton RD, Weaver CT (2012). Reciprocal interactions of the intestinal microbiota and immune system. Nature.

[2] Yatsunenko T, Rey FE, Manary MJ (2012). Human gut microbiome viewed across age and geography. Nature.

[3] Aagaard K, Ma J, Antony KM, Ganu R, Petrosino J, Versalovic J (2014). The placenta harbors a unique microbiome. Sci Transl Med.

[4] Cilieborg MS, Boye M, Sangild PT. Bacterial colonization and gut development in preterm neonates. Early Hum Dev. 2012. doi: 10.1016/j.earlhumdev.2011.12.027. 10.1016/j.earlhumdev.2011.12.027 22284985

[5] Dominguez-Bello CEK, Contreras M, Magris M, Hidalgo G, Fierer N, Knight R (2010). Delivery mode shapes the acquisition and structure of the initial microbiota across multiple body habitats in newborns. Proc Natl Acad Sci U S A.

[6] Azad MB, Konya T, Guttman DS, Field CJ, Chari RS, Sears MR, Becker AB, Scott JA, Kozyrskyj AL (2014). Impact of cesarean section delivery and breastfeeding on infant gut microbiota at one year of age. Allergy Asthma Clin Immunol.

[7] Adlerberth I (2008). Factors influencing the establishment of the intestinal microbiota in infancy. Nestle Nutr Work Ser Pediatr Progr.

[8] Adlerberth I, Wold AE (2009). Establishment of the gut microbiota in Western infants. Acta Paediatr.

[9] Fanaro S, Chierici R, Guerrini P, Vigi V (2003). Intestinal microflora in early infancy: composition and development. Acta Paediatr Suppl.

[10] Hesla HM, Stenius F, Jäderlund L, Nelson R, Engstrand L, Alm J, Dicksved J. Impact of lifestyle on the gut microbiota of healthy infants and their mothers—the Aladdin birth cohort. FEMS Microbiol Ecol. 2014. doi: 10.1111/1574-6941.12434. 10.1111/1574-6941.12434 25290507

[11] Hussey S, Wall R, Gruffman E, O’Sullivan L, Ryan CA, Murphy B, Fitzgerald G, Stanton C, Ross RP. Parenteral antibiotics reduce bifidobacteria colonization and diversity in neonates. Int J Microbiol. 2011. doi: 10.1155/2011/130574. 10.1155/2011/130574 PMC2929493 20811542

[12] Bäckhed F, Roswall J, Peng Y (2015). Dynamics and stabilization of the human gut microbiome during the first year of life. Cell Host Microbe.

[13] Tannock GW, Lawley B, Munro K (2013). Comparison of the compositions of the stool microbiotas of infants fed goat milk formula, cow milk-based formula, or breast milk. Appl Environ Microbiol.

[14] Koenig JE, Spor A, Scalfone N, Fricker AD, Stombaugh J, Knight R, Angenent LT, Ley RE (2011). Succession of microbial consortia in the developing infant gut microbiome. Proc Natl Acad Sci U S A.

[15] La Rosa PS, Warner BB, Zhou Y (2014). Patterned progression of bacterial populations in the premature infant gut. Proc Natl Acad Sci.

[16] Gale C, Logan KM, Santhakumaran S, Parkinson JRC, Hyde MJ, Modi N (2012). Effect of breastfeeding compared with formula feeding on infant body composition: a systematic review and meta-analysis. Am J Clin Nutr.

[17] Innis S (2007). Human milk: maternal dietary lipids and infant development. Proc Nutr Soc.

[18] Palmer C, Bik EM, DiGiulio DB, Relman DA, Brown PO (2007). Development of the human infant intestinal microbiota. PLoS Biol.

[19] Knol J, Scholtens P, Kafka C, Steenbakkers J, Gro S, Helm K, Klarczyk M, Schöpfer H, Böckler H-M, Wells J (2005). Colon microflora in infants fed formula with galacto- and fructo-oligosaccharides: more like breast-fed infants. J Pediatr Gastroenterol Nutr.

[20] Stewart CJ, Marrs ECL, Nelson A, Lanyon C, Perry JD, Embleton ND, Cummings SP, Berrington JE (2013). Development of the preterm gut microbiome in twins at risk of necrotising enterocolitis and sepsis. PLoS One.

[21] Groer MW, Luciano AA, Dishaw LJ, Ashmeade TL, Miller E, Gilbert JA (2014). Development of the preterm infant gut microbiome: a research priority. Microbiome.

[22] Kamada N, Núñez G (2014). Regulation of the immune system by the resident intestinal bacteria. Gastroenterology.

[23] Zeissig S, Blumberg RS (2014). Life at the beginning: perturbation of the microbiota by antibiotics in early life and its role in health and disease. Nat Immunol.

[24] Kamada N, Seo S-U, Chen GY, Núñez G (2013). Role of the gut microbiota in immunity and inflammatory disease. Nat Rev Immunol.

[25] Yu Z, Morrison M (2004). Improved extraction of PCR-quality community DNA from digesta and fecal samples. Biotechniques.

[26] Fernández-Peralbo M, de Castro L. Preparation of urine samples prior to targeted or untargeted metabolomics mass-spectrometry analysis. doi: 10.1016/j.trac.2012.08.011.

[27] Magoč T, Salzberg SL (2011). FLASH: fast length adjustment of short reads to improve genome assemblies. Bioinformatics.

[28] Edgar RC (2013). UPARSE: highly accurate OTU sequences from microbial amplicon reads. Nat Methods.

[29] Wang Q, Garrity GM, Tiedje JM, Cole JR (2007). Naive Bayesian classifier for rapid assignment of rRNA sequences into the new bacterial taxonomy. Appl Environ Microbiol.

[30] Benjamini Y, Hochberg J (1995). Controlling the false discovery rate: a practical and powerful approach to multiple testing. J R Stat Soc Ser B.

[31] Langfelder P, Horvath S (2008). WGCNA: an R package for weighted correlation network analysis. BMC Bioinformatics.

[32] Claesson MJ, Jeffery IB, Conde S (2012). Gut microbiota composition correlates with diet and health in the elderly. Nature.

[33] Azad MB, Konya T, Maughan H, Guttman DS, Field CJ, Chari RS, Sears MR, Becker AB, Scott JA, Kozyrskyj AL (2013). Gut microbiota of healthy Canadian infants: profiles by mode of delivery and infant diet at 4 months. CMAJ.

[34] Avershina E, Storrø O, Øien T, Johnsen R, Wilson R, Egeland T, Rudi K (2013). Bifidobacterial succession and correlation networks in a large unselected cohort of mothers and their children. Appl Environ Microbiol.

[35] Dogra S, Sakwinska O, Soh S-E (2015). Dynamics of infant gut microbiota are influenced by delivery mode and gestational duration and are associated with subsequent adiposity. MBio.

[36] Hyde MJ, Griffin JL, Herrera E, Byrne CD, Clarke L, Kemp PR (2010). Delivery by Caesarean section, rather than vaginal delivery, promotes hepatic steatosis in piglets. Clin Sci.

[37] Dessì A, Atzori L, Noto A (2011). Metabolomics in newborns with intrauterine growth retardation (IUGR): urine reveals markers of metabolic syndrome. J Matern Fetal Neonatal Med.

[38] Russell WR, Duncan SH, Scobbie L, Duncan G, Cantlay L, Calder AG, Anderson SE, Flint HJ (2013). Major phenylpropanoid-derived metabolites in the human gut can arise from microbial fermentation of protein. Mol Nutr Food Res.

[39] O’Mahony SM, Clarke G, Borre YE, Dinan TG, Cryan JF (2014). Serotonin, tryptophan metabolism and the brain-gut-microbiome axis. Behav Brain Res.

[40] Zheng P, Zeng B, Zhou C, et al. Gut microbiome remodeling induces depressive-like behaviors through a pathway mediated by the host’s metabolism. Mol Psychiatry. 2016;21(6):786–96. 10.1038/mp.2016.44 27067014

[41] Strandvik B, Wahlén E, Wikström S-A (1994). The urinary bile acid excretion in healthy premature and full-term infants during the neonatal period. Scand J Clin Lab Invest.

[42] Yousef IM, Perwaiz S, Lamireau T, Tuchweber B (2003). Urinary bile acid profile in children with inborn errors of bile acid metabolism and chronic cholestasis; screening technique using electrospray tandem mass-spectrometry (ES/MS/MS). Med Sci Monit.

[43] Weuve J, Sánchez BN, Calafat AM, Schettler T, Green RA, Hu H, Hauser R (2006). Exposure to phthalates in neonatal intensive care unit infants: urinary concentrations of monoesters and oxidative metabolites. Environ Health Perspect.

[44] Carlstedt F, Jönsson BAG, Bornehag C-G (2013). PVC flooring is related to human uptake of phthalates in infants. Indoor Air.

[45] Koeth RA, Wang Z, Levison BS (2013). Intestinal microbiota metabolism of L-carnitine, a nutrient in red meat, promotes atherosclerosis. Nat Med.

[46] Marcobal A, Barboza M, Sonnenburg ED (2011). Bacteroides in the infant gut consume milk oligosaccharides via mucus-utilization pathways. Cell Host Microbe.

[47] Murphy K, O’ Shea CA, Ryan CA, Dempsey EM, O’ Toole PW, Stanton C, Ross RP (2015). The gut microbiota composition in dichorionic triplet sets suggests a role for host genetic factors. PLoS One.

[48] Cox LM, Yamanishi S, Sohn J (2014). Altering the intestinal microbiota during a critical developmental window has lasting metabolic consequences. Cell.

[49] van Nimwegen FA, Penders J, Stobberingh EE (2011). Mode and place of delivery, gastrointestinal microbiota, and their influence on asthma and atopy. J Allergy Clin Immunol.

[50] Kerr CA, Grice DM, Tran CD, Bauer DC, Li D, Hendry P, Hannan GN (2014). Early life events influence whole-of-life metabolic health via gut microflora and gut permeability. Crit Rev Microbiol.

[51] Rodríguez JM, Murphy K, Stanton C (2015). The composition of the gut microbiota throughout life, with an emphasis on early life. Microb Ecol Health Dis.

[52] Turnbaugh PJ, Hamady M, Yatsunenko T (2009). A core gut microbiome in obese and lean twins. Nature.

[53] Nicholson JK, Holmes E, Kinross J, Burcelin R, Gibson G, Jia W, Pettersson S (2012). Host-gut microbiota metabolic interactions. Science.

[54] Clarke G, O’Mahony SM, Dinan TG, Cryan JF (2014). Priming for health: gut microbiota acquired in early life regulates physiology, brain and behaviour. Acta Paediatr.

[55] Bezirtzoglou E, Tsiotsias A, Welling GW (2011). Microbiota profile in feces of breast- and formula-fed newborns by using fluorescence in situ hybridization (FISH). Anaerobe.

[56] Yamasaki C, Totsu S, Uchiyama A, Nakanishi H, Masumoto K, Washio Y, Shuri K, Ishida S, Imai K, Kusuda S (2012). Effect of Bifidobacterium administration on very-low-birthweight infants. Pediatr Int.

[57] Saez-Lara MJ, Gomez-Llorente C, Plaza-Diaz J, Gil A. The role of probiotic lactic acid bacteria and bifidobacteria in the prevention and treatment of inflammatory bowel disease and other related diseases: a systematic review of randomized human clinical trials. Biomed Res Int. 2015;2015:505878. 10.1155/2015/505878 PMC4352483 25793197

[58] Picard C, Fioramonti J, Francois A, Robinson T, Neant F, Matuchansky C (2005). Review article: bifidobacteria as probiotic agents—physiological effects and clinical benefits. Aliment Pharmacol Ther.

[59] Solís G, de Los Reyes-Gavilan CG, Fernández N, Margolles A, Gueimonde M (2010). Establishment and development of lactic acid bacteria and bifidobacteria microbiota in breast-milk and the infant gut. Anaerobe.

[60] Ahrne S, Lonnermark E, Wold AE, Aberg N, Hesselmar B, Saalman R, Strannegard IL, Molin G, Adlerberth I (2005). Lactobacilli in the intestinal microbiota of Swedish infants. Microbes Infect.

