# Erratum to: Evolution of gut microbiota composition from birth to 24 weeks in the INFANTMET Cohort

**DOI:** 10.1186/s40168-017-0240-3

**Published:** 2017-02-14

**Authors:** Cian J. Hill, Denise B. Lynch, Kiera Murphy, Marynka Ulaszewska, Ian B. Jeffery, Carol Anne O’Shea, Claire Watkins, Eugene Dempsey, Fulvio Mattivi, Kieran Tuohy, R. Paul Ross, C. Anthony Ryan, Paul W. O’Toole, Catherine Stanton

**Affiliations:** 1 School of Microbiology, University College Cork, Cork, Ireland; 2 APC Microbiome Institute, University College Cork, Cork, Ireland; 3 Teagasc Moorepark Food Research Centre, Fermoy, Co., Cork, Ireland; 4 Department of Neonatology, Cork University Maternity Hospital, Cork, Ireland; 5 Food Quality and Nutrition Department, Research and Innovation Centre, Fondazione Edmund Mach, San Michele All′adige, Italy

## Erratum

Following publication of this article [1], it has come to our attention that the name of the author Kieran Tuohy’s name was captured incorrectly as “Touhy” and instead should be Kieran Tuohy.
