# Supplementary material for: Evolution of gut microbiota composition from birth to 24 weeks in the INFANTMET Cohort
Source: Microbiome. 2017 Jan 17;5:4. doi: 10.1186/s40168-016-0213-y (PMC5240274; doi:10.1186/s40168-016-0213-y)
Supplement: Supplementary file 2 — Supplementary Materials and Methods. (DOC 39 kb) [file 40168_2016_213_MOESM2_ESM.doc]

## Supplementary Materials and Methods

## Metabolomics analysis of infant urine

**Chemical and reagents**

HPLC-grade methanol, acetonitrile, and formic acid were obtained from Sigma Aldrich. The ultrapure water was obtained by purifying demineralized water in a Milli-Q system from Millipore (Bedford, MA, USA). Internal standards creatinine labeled ^13^C was purchased from Sigma Aldrich and d_5_ labeled trans-cinnamic acid, hippuric acid-d5, tryptophan-d5, cholic acid-d4, deoxycholic acid-2,2,4,4-d4, ursodeoxycholic acid-2,2,4,4-d4, lithocholic acid-d4, chenodeoxycholic acid-d4, glycochenodeoxycholic acid-2,2,4,4-d4, glycolithocholic acid-2,2,4,4-d4, glycoursodeoxycholic acid-d4 , glycocholic acid-d4, taurochenodeoxycholic acid-d5, taurolithocholic acid d5 sodium salt, taurocholic acid-d5 sodium salt, taurodeoxycholic-2,2,3,4,4-d5 acid, acetyl-L-carnitine-d3, L-carnitine d3, propionyl-L-carnitine-d3, butyryl-L-carnitine-d3, octanoyl-L-carnitine-d3, decanoyl-L-carnitine-d3, dodecanoyl-L-carnitine-d3,tetradecanoyl-L-carnitine-d3,hexadecanoyl-L-carnitine-d3, octadecanoyl-L-carnitine-d3 were obtained from CDN ISOTOPES, Inc. (Pointe-Claire, Quebec, Canada). PVDF syringe filters 0.45 µm were obtained from Millipore.

**Chromatographic and mass spectrometric conditions**

A hybrid linear ion trap Fourier Transform (LTQ FT) Orbitrap mass spectrometer (Thermo Fisher, Bremen, Germany) was interfaced to a Dionex HPLC system, consisting of an autosampler and quaternary gradient HPLC-pump. Chromatographic separation of the compounds was performed using a Kinetex C18 column (150 mm × 2.1 mm I.D., particle size 3.5 µm) (Phenomenex Torrance, CA, USA). The pre-column used was a 4.0 mm × 2.0 mm I.D. Phenomenex Security Guard column. The analytical and the guard columns were maintained at a temperature of 30 ^o^C in a column thermostat. An optimized gradient was used at a constant flow rate of 0.3 mL /min using Milli-Q water (Solvent A) and acetonitrile (Solvent B) both with 0.1% formic acid. The percentage of organic modifier (B) was changed linearly as follows: 0 min, 5%; 20 min, 100%; 30 min, 100%; 32 min, 5%. Between consecutive runs, the analytical column was re-equilibrated for 10 min.

The Orbitrap LTQ was equipped with an Electrospray Ionization (ESI) probe and operated in the positive and negative ionization modes. The conditions in ESI positive (and negative) mode were: source voltage 5.0 kV (3.5 kV), heated capillary temperature 320 ◦C, capillary voltage 30 V (-30 V) and tube lens 110 V (-110 V). In the LTQ component of the instrument, nitrogen was used as both the sheath gas (70 U) and auxiliary gas (30 U), and helium was used as the damping gas. All measurements were done using the automatic gain control (AGC) of the LTQ to adjust the number of ions entering the trap. Samples were injected two times per each ionization assay. In positive ionization mode one injection was performed in full scan mode only at mass resolution 30,000 in centroid mode, while during the second injection mass spectrometer operated under data-dependent-acquisition (DDA) mode during the complete chromatographic run, in which both MS and MS^n^ spectra were acquired simultaneously. The resolving power for MS^2^ scans was 7500. Product ions were generated in the LTQ trap at collision energy 35 eV using an isolation width of 1 Da.

Mass calibration was performed with every sequence run just prior to starting the batch by using flow injection of the manufacturer’s calibration standards mixture allowing for mass accuracies <5 ppm in external calibration mode. 10 µL of sample were injected to the system. The static exclusion list was made up of the 300 most abundant ions created by the injection of solvents, which followed the same preparation procedures as the samples. Dynamic exclusion allowed 2 repeated counts of the same ion in 15 s, while the exclusion duration was 45 s. The sequences were randomised, every 15 samples a quality control block was analysed consisting of solvents, internal standards and two quality control samples

**Statistical Analysis and Biomarker Identification**

Data received from untargeted mass spectrometry based assays were converted from .raw to mzXML with the msconvert utility included in ProteoWizard (Chambers et al. 2012). Downstream, profiling data was processed with XCMS on-line tool (Smith et al. 2006) (https://xcmsonline.scripps.edu/) using the ‘‘centWave’’ peak picking method. The minimum signal to noise ratio was set to 5, while the ppm to 20. XCMS online provides a solution for the complete untargeted metabolomic workflow including m/z feature detection, retention time correction, alignment, annotation, and data visualization. Data obtained from this processing consisted of list of m/z features and its relative intensities, which vary between sample groups. Such matrix file, with information about sample codes, m/z feature and its intensity, was used for statistical analysis.

The m/z features that were found to be statistically significant and contributed to the discrimination between groups were identified through a multiple-step procedure. CAMERA was used for identification of clusters of ions originating from the same metabolite (i.e., molecular ions, adducts and 13C isotopes). Additionally in-source molecular fragments were assigned based on mass accuracy approach, as many of them were statistically significant. Most common observed fragments corresponded to loss of -SO3 (-79.9568 Da), -CO2 (-43.9898 Da), -H2O (18.0106 Da) and -C2H4O (- 44.0262 Da) , -C6H8O6 (-176.0320 Da). Discriminative markers were then compared with the monoisotopic molecular weight, chemical structures and LC-MS/MS spectra of metabolites proposed by freely available databases: m/z Cloud (www.mzcloud.org); the Human Metabolome DB (Wishart et al. 2009), the METLIN (Smith et al. 2005), the MassBank (Horai et al. 2010) and the LIPID MAPS (Sud et al. 2010) databases. Mass accuracy was set to 2 mDa while searching on-line. Additionally, information from MSn experiments were introduced to Met-Fusion to get candidate structures (Gerlich and Neumann 2013). Final identification was achieved after a combination of LC-HRMS^2^, LC-HRMS^3^ experiments, on-line database information and literature verification. Levels of identification were as follow: Level I corresponds to compounds identified by matching masses and retention times with authentic standards in the laboratory, or by matching with LC-HR-MS and LC-HR MS/MS and LC-HR-MSn of standards reported in the literature; Level II corresponds to compounds identified by LC-MS, LC-MS2 and/or LC-MSn spectra and matched to spectra from databases and literature. Compounds identified only by spectral similarities to a similar compound class and literature knowledge are reported as level III. Unknown compounds are reported as level IV. Full information is available in Supp. Table 17.
