# Supplementary figures and images for: Evolution of gut microbiota composition from birth to 24 weeks in the INFANTMET Cohort
Source: Microbiome. 2017 Jan 17;5:4. doi: 10.1186/s40168-016-0213-y (PMC5240274; doi:10.1186/s40168-016-0213-y)

# Spearman distances between twins and non-twins

Week 1

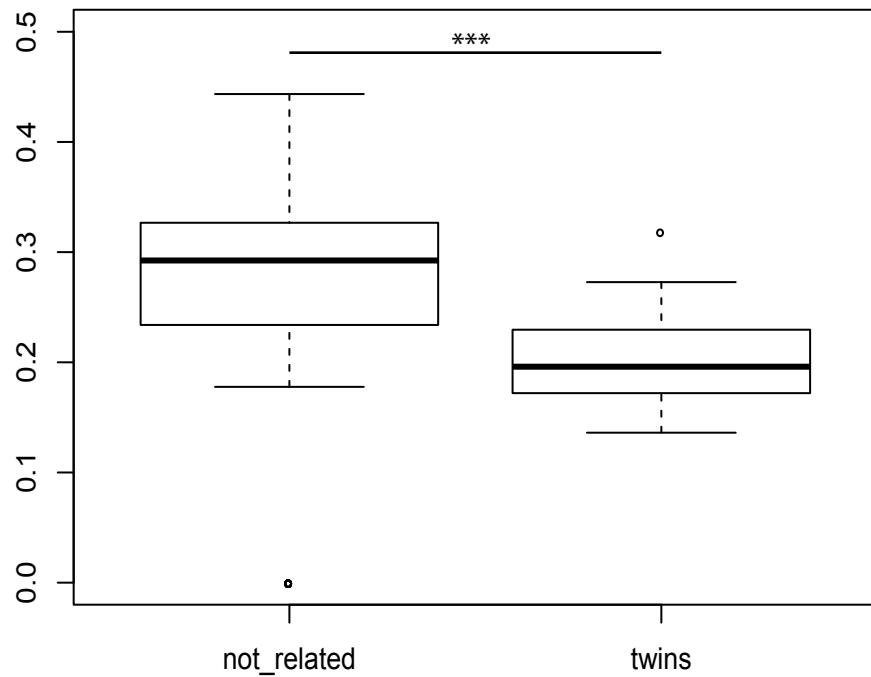

Week 4

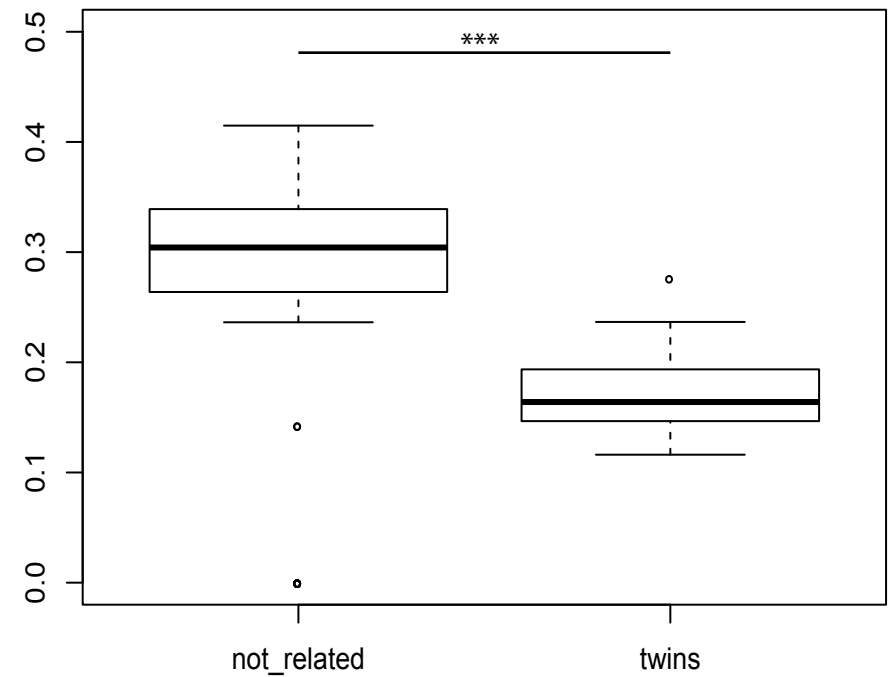

Week 8

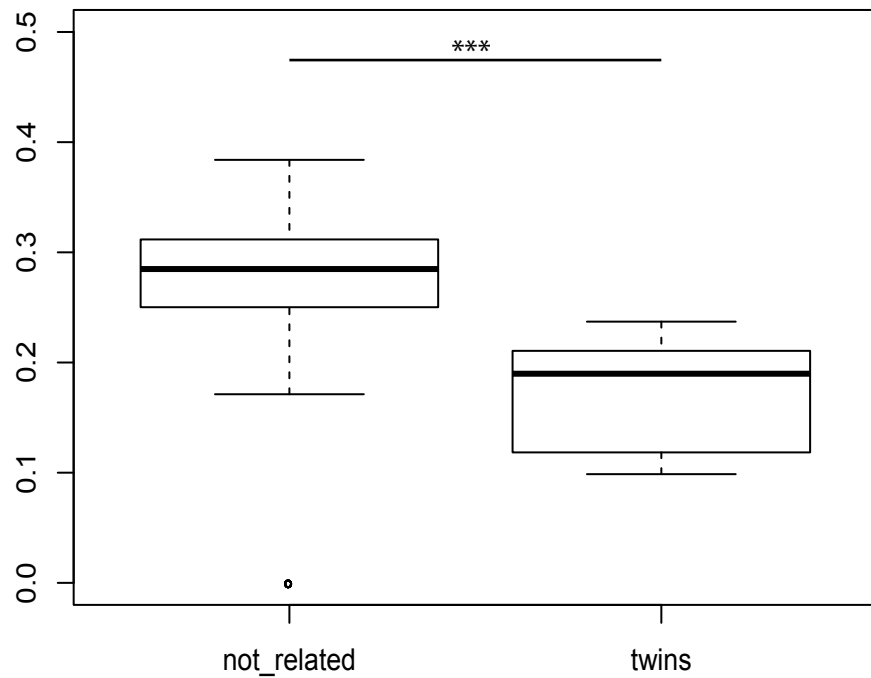

Week 24

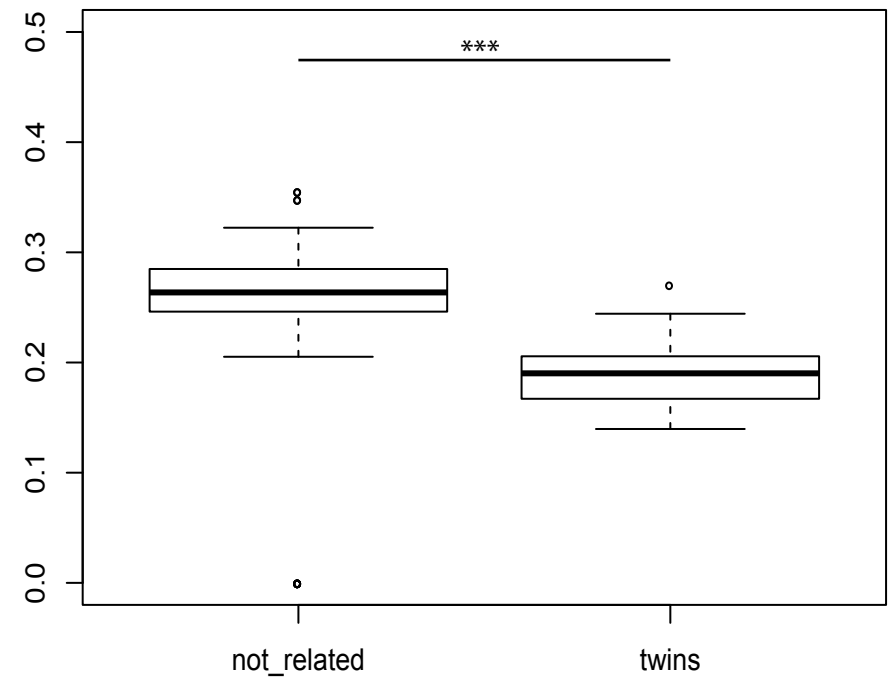

Supplement: Supplementary file 4 — Twins have more similar microbiota to one another than an unrelated infant at 1, 4, 8, and 24 weeks of age. Using t tests with Monte-Carlo permutations, the Spearman distance between twins’ microbiota was determined and compared to unrelated infants’ microbiota (***p < 0.001). (PDF 138 kb) [file 40168_2016_213_MOESM4_ESM.pdf]

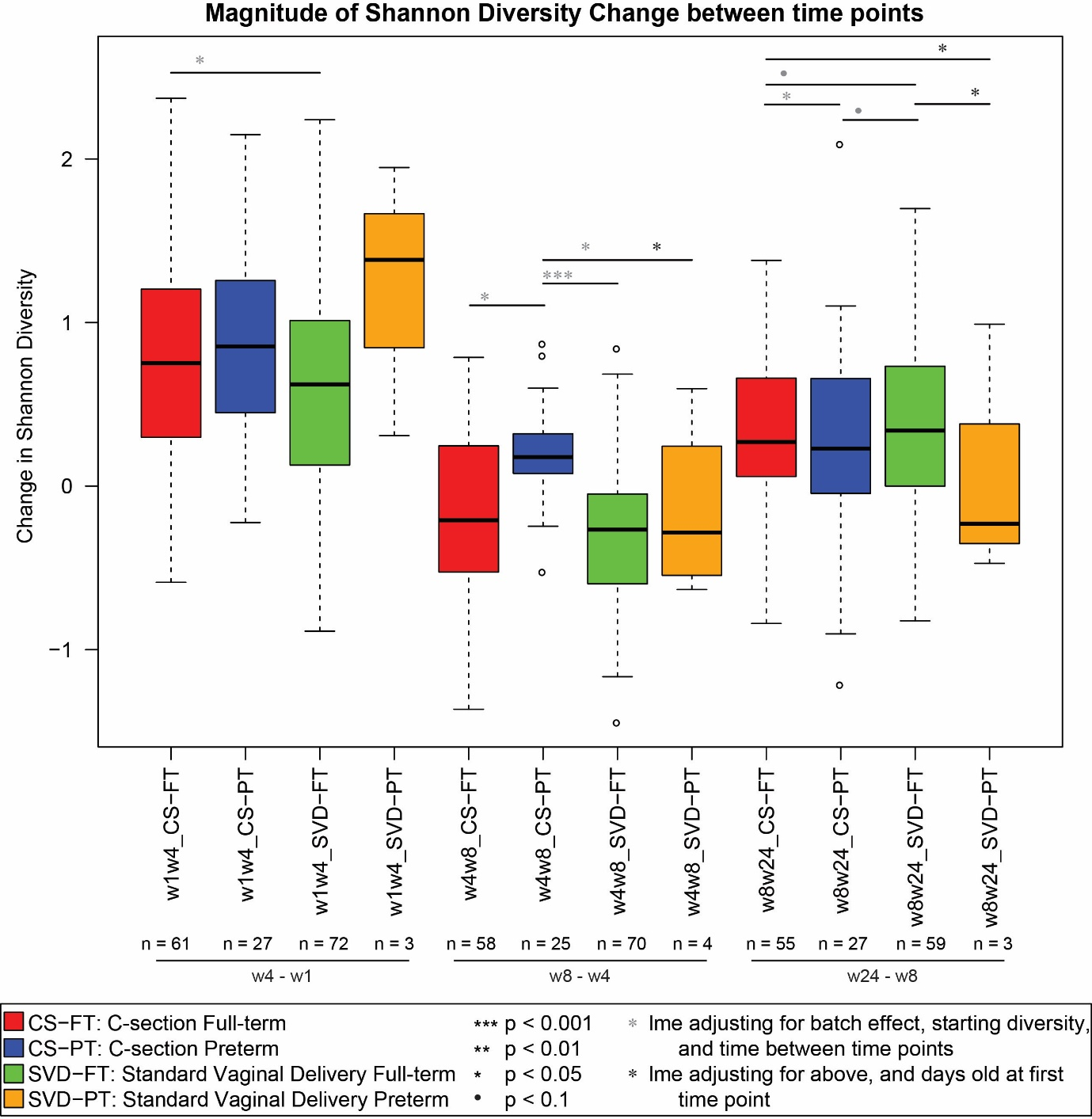

Supplement: Supplementary file 5 — Assessing the magnitude of change of Shannon diversity between time points for subjects of a given birth mode. Statistical models were used to determine if infants of one birth mode changed more or less than infants of other birth modes at different stages. Grey asterisks highlight significant differences from linear mixed effects models that adjust for batch effect and the number of days between time points for each subject. Black asterisks highlight significant differences as determined by linear mixed effects models, adjusting for batch effect, the number of days between time points, and the age of infants at the first time point. (TIF 638 kb) [file 40168_2016_213_MOESM5_ESM.tif]
