# Supplementary material for: Evolution of gut microbiota composition from birth to 24 weeks in the INFANTMET Cohort
Source: Microbiome. 2017 Jan 17;5:4. doi: 10.1186/s40168-016-0213-y (PMC5240274; doi:10.1186/s40168-016-0213-y)
Supplement: Supplementary file 3 — A selection of specific metabolites driving the split in the co-inertia analysis that separates FT and PT gut by their metabolomic profile. Each metabolite is represented by one or more features. The metabolites on the left hand side are higher in PT infants and the metabolites on the right hand side are higher in FT infants. (PDF 21785 kb) [file 40168_2016_213_MOESM3_ESM.pdf]

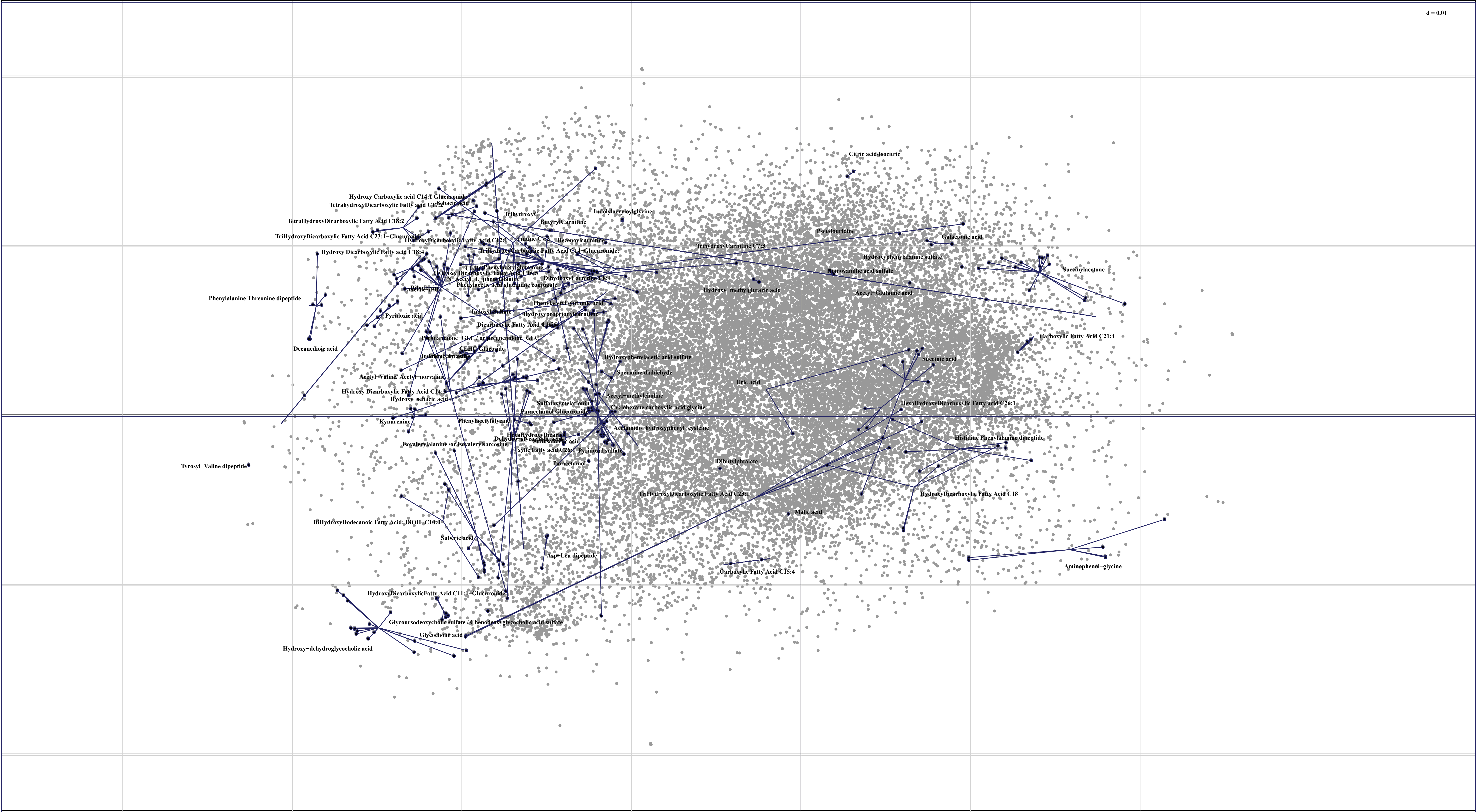
d = 0.01
Citric acid/Isocitric
Hydroxy Carboxylic acid C14:1 Glucuronide
TetraHydroxyDicarboxylic Fatty Acid C18:2
TriHydroxyDicarboxylic Fatty Acid C23:1−Glucuronide
Indolylacryloylglycine
ButyrylCarnitine
Decenoylcarnitine
Pseudouridine
Galactonic acid
TrihydroxyCarnitine C7:3
TriHydroxyCarboxylic Fatty Acid C14−Glucuronide
Hydroxy Dicarboxylic Fatty acid C18:4
Hydroxyphenylalanine sulfate
Succinylacetone
Phenylacetylglutamine
Homovanillic acid sulfate
DihydroxyCarnitine C8:4
Phenylacetic acid glutamine conjugate
Azelaic acid
Phenylalanine Threonine dipeptide
Hydroxy−methylglutaric acid
Acetyl−Glutamic acid
Phenylacetylglutamic acid
Pyridoxic acid
Indoxyl sulfate
Hydroxypropionylcarnitine
Decanedioic acid
Pregnanedione−GLC / or pregnenolone−GLC
Carboxylic Fatty Acid C21:4
CEHC Glucoside
Succinic acid
Hydroxyphenylacetic acid sulfate
Spermine dialdehyde
Uric acid
Acetyl−Valine/ Acetyl−norvaline
Hydroxy−sebacic acid
Acetyl−methylcholine
HexaHydroxyDicarboxylic Fatty acid C26:1
Sulfatoxymelatonin
Paracetamol Glucuronide
Cyclohexane carboxylic acid glycine
Kynurenine
Phenylacetylglycine
Acetamido−hydroxyphenyl−cysteine
Histidine Phenylalanine dipeptide
isovalerylalanine /or isovalerylsarcosine
Pyridoxal sulfate
Tyrosyl−Valine dipeptide
Paracetamol
Dibutylphtalate
TriHydroxyDicarboxylic Fatty Acid C23:1
HydroxyDicarboxylic Fatty Acid C18
Malic acid
DiHydroxyDodecanoic Fatty Acid: DiOH−C10:0
Suberic acid
Asp−Leu dipeptide
Carboxylic Fatty Acid C15:4
Aminophenol−glycine
HydroxyDicarboxylicFatty Acid C11:1−Glucuronide
Glycoursodeoxycholic sulfate / Chenodeoxyglycocholic acid sulfate
Glycocholic acid
Hydroxy−dehydroglycocholic acid
